# Microvertebrate faunal assemblages of the Favel Formation (late Cenomanian-middle Turonian) of Manitoba, Canada

**DOI:** 10.7717/peerj.15493

**Published:** 2023-08-03

**Authors:** Aaron A. Kilmury, Aaron Anderson, Dhuranka S. Wijesinghe, Ashley F. Verstraete, William Ezeana, Ashley E. Anderson, Kirstin S. Brink

**Affiliations:** Earth Sciences, University of Manitoba, Winnipeg, Manitoba, Canada

**Keywords:** Fossil, Marine, Fish, Shark, Reptile, Bird, Cretaceous, Biogeography, Paleoenvironment, Stratigraphy

## Abstract

Microvertebrate assemblages of the Upper Cretaceous (late Cenomanian to mid-Turonian) Favel Formation of Manitoba are formally described for the first time. New vertebrate occurrences from the Favel Formation include the actinopterygians Caturidae indet., cf. Albulidae *incertae sedis*, *Micropycnodon kansasensis*, *Pachyrhizodus minimus*, *Protosphyraena* sp., *Thryptodus loomisi*, chondrichthyans *Ischyrhiza* cf. *mira*, *I. texana*, *Ptychodus marginalis*, *P. occidentalis*, and *P. rhombodus*, the avian cf. *Ichthyornis* sp., the reptile Testudines indet., and an unknown taxon referred to as Vertebrate A. Changes in faunal occurrences throughout the formation suggest an offshore open marine environment for the lower and middle horizons and nearshore marine for the upper horizon, represent ing mid- and late stages of the Greenhorn third-order marine cycle. This newly described diversity increases biogeographic affinities of the late Cenomanian to mid-Turonian vertebrate assemblages of Manitoba with central WIS localities in South Dakota and Kansas, providing additional support for a central vertebrate biogeographic subprovince during late Cenomanian to early Turonian times, as well as WIS localities further south in Texas decreasing the gradient of the north-south or central-south community boundary during early and mid-Turonian times.

## Introduction

The Favel Formation of Manitoba (MB) represents a succession of offshore marine sediments deposited during the late Cenomanian to mid-Turonian Greenhorn marine cycle of the Western Interior Seaway (WIS) and includes calcareous mudstones, lime packstones and wackestones, calcarenites, and multiple series of bentonite beds (McNeil & Caldwell, 1981; Nicolas, Bamburak & Hosseininejad, 2013).

Globally recognized events representing significant climate changes recorded in the Favel Formation include the Cenomanian-Turonian oceanic anoxic event (OAE-2), the associated Cretaceous Thermal Maximum, and peak transgression of the Greenhorn WIS marine cycle (Kyser et al., 1993; Schröder-Adams, 2014; Dionne, Schröder-Adams & Cumbaa, 2016; Scotese et al., 2021). These events have been recognized in the form of δ^18^O trends analyzed from phosphate of fish remains, δ^18^O and δ^13^C trends from calcareous planktonic foraminifers and coccoliths, aragonite of ammonites, aragonite and calcite prisms of inoceramid clams, δ^13^C, organic carbon, and hydrogen index trends from mudstones, as well as relative abundances of foraminifera (Kyser et al., 1993; Schröder-Adams, 2014; Dionne, Schröder-Adams & Cumbaa, 2016; Scotese et al., 2021). In the Riding Mountain area of MB, the stratigraphic position of the pronounced positive organic δ^13^C excursion associated with the OAE2 event is approximately 8 to 9 m above the Ashville-Favel interformational contact and near the base of the Laurier Limestone beds of the Keld Member (Kyser et al., 1993). The Laurier Limestone beds represent a series of 5 to 15 cm thick argillaceous limestone beds within the uppermost 5 m of the Keld Member, with the top of the uppermost limestone bed as the contact with the overlying Assiniboine Member, and were deposited during peak transgression of the Greenhorn Sea (McNeil & Caldwell, 1981; Kyser et al., 1993). The significant changes in both environmental conditions represented by prolonged excursions in δ^18^O and δ^13^C trends and depositional environments represented by series of mudstone and limestone beds likely had drastic impacts on the marine life that inhabited these environments and are recorded in the fossil record of the Favel Formation.

Paleontology of the Favel Formation has been previously investigated and described in terms of macrovertebrates (Bardack, 1968), macroinvertebrates (Wickenden, 1945), foraminifera (McNeil & Caldwell, 1981; Schröder-Adams et al., 2001; Dionne, Schröder-Adams & Cumbaa, 2016), and nannofossils (Schröder-Adams et al., 2001); however, microvertebrate fossil occurrences have not yet been described in detail. Although several geologists provided early reports of vertebrate fossils from the Manitoba escarpment, Bardack (1968) was the first to provide descriptions, including descriptions of the first known vertebrate fossils collected in present-day MB, two chondrichthyan symphyseal teeth (CMN 5071) collected by J.W. Spencer in 1874 and assigned to *Ptychodus parvulus* by Whiteaves (1889), though they cannot be identified to a particular species based on their morphologies, and two chondrichthyan tooth occurrences presumably from the Marco Calcarenite near the top of the Favel Formation (Kirk, 1930).

Previous work describing Upper Cretaceous microvertebrate assemblages of the MB escarpment is limited to the mid-Cenomanian portion of the Belle Fourche Member, Ashville Formation in Saskatchewan (Schröder-Adams et al., 2001; Cumbaa, Shimada & Cook, 2010). Although microvertebrate fossil horizons have been noted from Upper Cretaceous units other than the Belle Fourche Member (McNeil & Caldwell, 1981; Bamburak, Hatcher & Nicolas, 2012; Nicolas, Bamburak & Hosseininejad, 2013), descriptions of their faunal assemblages are lacking and their underrepresentation in museum fossil collections introduces a significant size bias when comparing micro- and macrovertebrate occurrences (Kilmury, Nicolas & Brink, 2021). As shown by descriptions of mid-Cenomanian microvertebrates of Saskatchewan, microvertebrate assemblages contain a wealth of information useful in representing small-bodied and endemic vertebrate taxa, as well as interpreting depositional, environmental, and habitat conditions, and biogeographic affinities (Schröder-Adams et al., 2001; Cumbaa et al., 2006; Cumbaa, Shimada & Cook, 2010; Underwood & Cumbaa, 2010).

Here, three fossiliferous stratigraphic horizons from the lower, middle, and upper Favel Formation, sampled from four sites, are described in terms of their microvertebrate fossil content, faunal compositions, and biogeographic affinities, in order to gain an improved understanding of vertebrate biostratigraphy and the marine communities and habitats represented by Favel Formation fossil assemblages.

## Methods

**Figure 1:**
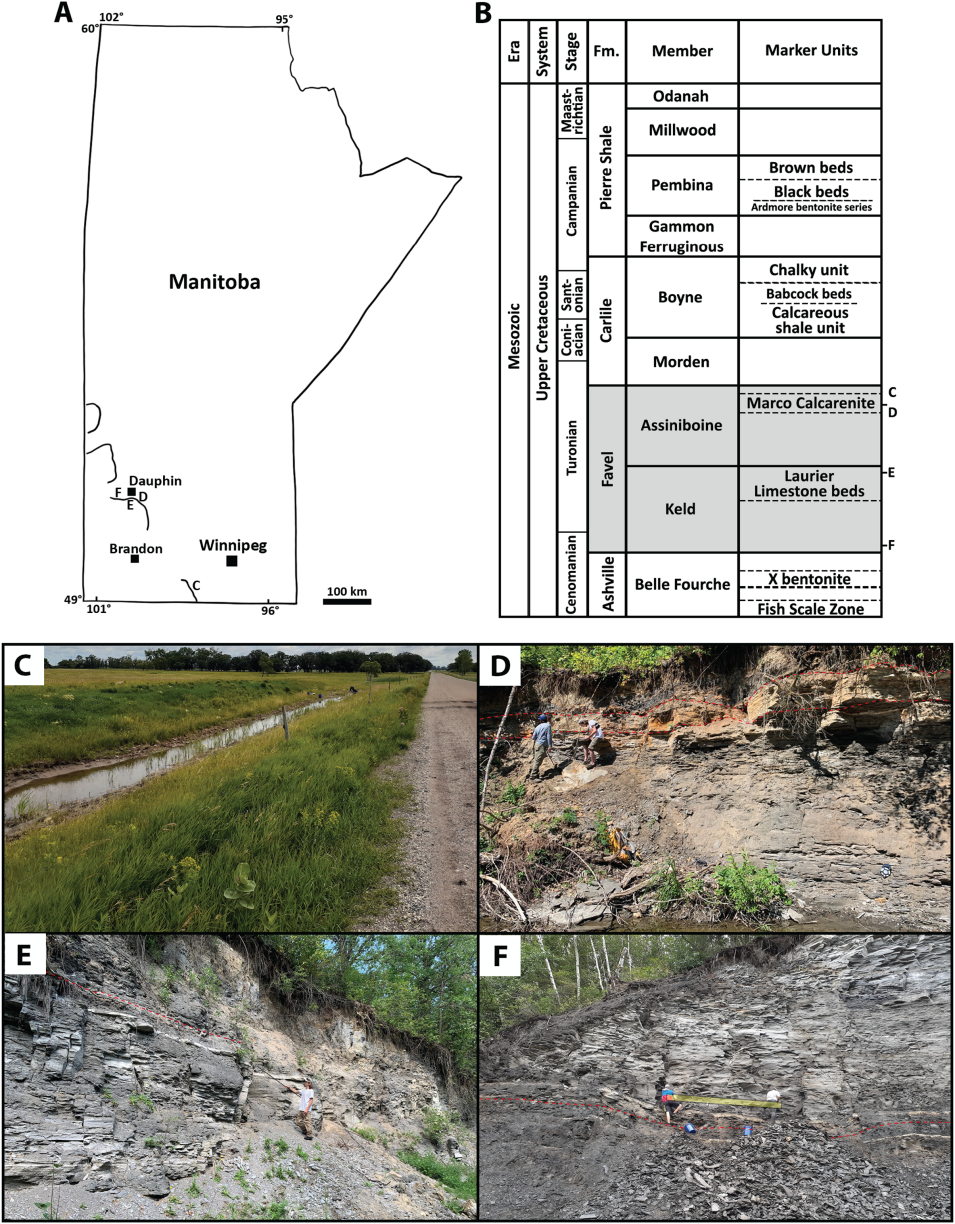
Sites and horizons sampled for microvertebrate analyses. (A) Simplified map of Manitoba showing general locations of sites: C) ‘Marco Calcarenite, near Miami’, D) ‘Marco Calcarenite, Ochre R.’, E) ‘Laurier Limestone beds, Edwards Creek’, and F) ‘Near Keld base, Vermilion R.’, with lines representing topographically high portions of the Manitoba escarpment. (B) Lithostratigraphic chart showing the Upper Cretaceous units of Manitoba and relative stratigraphic positions of Favel Formation (shaded) sampled horizons (D–F). Lithostratigraphic units are not to scale and age determinations are in reference to McNeil & Caldwell (1981) and Bamburak, Hamilton & Heaman (2016). (C) Outcrop image of ‘Marco Calcarentie, near Miami’. Scale: Outcrop exposure is approximately 2 m wide. (D) Outcrop image of ‘Marco Calcarenite, Ochre R.’. Red dashed lines indicate the bottom and top of the Marco Calcarenite. Scale: person on left is 1.85 m in height. (E) Outcrop image of ‘Laurier Limestone beds, Edwards Creek’. Red dashed lines indicate the top of the Laurier Limestone beds and Keld-Assiniboine contact. Sampled horizons are pointed to with black pole. Scale: person is 1.72 m in height. (F) Outcrop image of ‘Near Keld base, Vermillion R.’. Red dashed line indicates the Ashville-Favel contact. Sampled horizons are indicated by the yellow rectangle. Scale: blue pail on right in 0.36 m in height.

**Figure 2:**
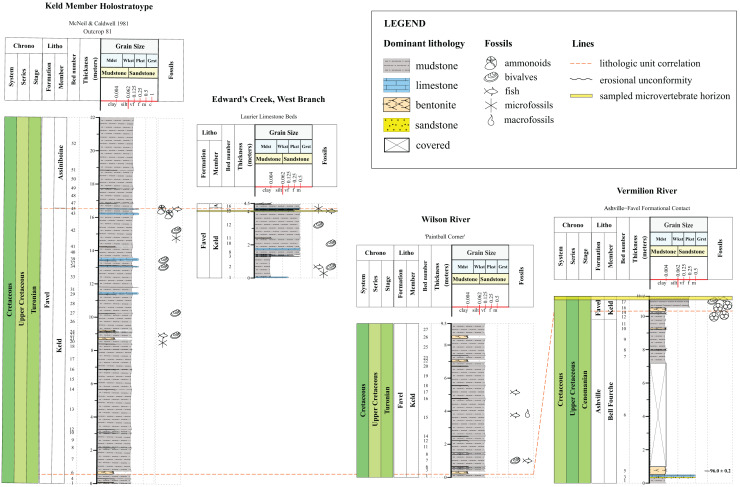
Lithostratigraphic correlations between the Assiniboine Member, Favel Formation holostratotype (Outcrop 80 in McNeil & Caldwell, 1981) and outcrop sections measured in the Dauphin area in 2020 and 2021. Stratigraphic position of the ‘Marco Calcarenite, Ochre R.’ microvertebrate horizon is shown in the ‘Skane’s Crossing, ‘Upstream Outcrop 1’ section (Fig. 1D), which is also the same outcrop where a bentonite was sampled and determined to have a 92.54 0.28 Ma radiometric age (Bamburak, Hamilton & Heaman, 2016).

**Figure 3:**
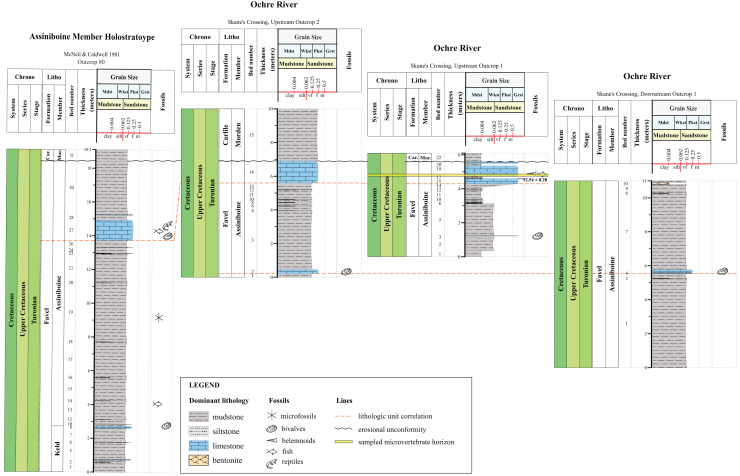
Lithostratigraphic correlations between the Keld Member, Favel Formation holostratotype (Outcrop 81 in McNeil & Caldwell, 1981) and outcrop sections measured in the Dauphin area in 2020 and 2021. Stratigraphic positions of the ‘Laurier Limestone bed, Edwards Creek’ and ‘Near Keld Base, Vermilion R.’ microvertebrate horizons are shown in the ‘Edwards Creek, West Branch’ and ‘Vermilion River’ sections, respectively. The 96.0 ± 0.2 Ma radiometric age shown in the ‘Vermilion River’ section beside the 47 cm thick bentonite, the “X” bentonite, was determined from a sample collected from the “X” bentonite 17.65 km northwest from the ‘Vermilion River’ section, along Wilson R. (Bamburak, Hamilton & Heaman, 2016).

Bulk rock samples collected for acid digestion and microvertebrate fossil analyses were sampled from 10–30 cm thick intervals of three stratigraphic horizons at four sites: ‘Marco Calcarenite, near Miami’, ‘Marco Calcarenite, Ochre R.’, ‘Laurier Limestone beds, Edwards Creek’, and ‘Near Keld base, Vermilion R.’ (Fig. 1). Sites sampled in the Dauphin area were chosen based on lithostratigraphic descriptions by McNeil & Caldwell (1981), previous work by the Manitoba Geological Survey (Nicolas, Bamburak & Hosseininejad, 2013), and previous fossil discoveries (Bardack, 1968). Sites were surveyed to assess stratigraphic position and fossil content prior to sample collection. Stratigraphic sections (Figs. 2, 3) recorded from bulk-sampled and nearby Favel Formation exposures in the Dauphin area were recorded at cm scale and digital columns created in RStudio using the SDAR package (Ortiz, Jaramillo & Moreno, 2020). Bulk rock samples and macrofossils collected by surface prospecting were collected under MB heritage permits A41-20 and A36-21.

Between 63 and 110 kg of bulk rock samples were collected from each site, 25–40 kg of which were digested in plastic bins, beginning with 3 L bins then upscaling to 33 L bins, using 10–20% acetic acid solution buffered with calcium phosphate. A Thermo Scientific pH meter (Thermo Fisher Scientific, Waltham, MA, USA) was used to measure pH of acid solutions and measured starting pH of 2.5–2.8 which increased to 4.1–4.7 after 3–4 weeks. After several months of digesting bulk rock samples in motionless acetic acid, submersible aquarium pumps (model GD034B) were suspended in each 33 L acid bin and room temperature was increased from ~21 °C to ~24 °C to accelerate acid digestion rates. Acid-resistant sediment and phosphatic fossil elements were recovered on a bi-weekly to monthly basis by first removing remaining large rock pieces and carefully removing solution from the top of bins without disturbing sediment at the bottom, slowly tilting bins and pouring in water to concentrate sediment to one side, slowly pouring sediment and water through several sediment sorting screens (0.25 to 6.73 mm), and then allowing the sediment to dry for at least 1 day before examination under dissecting microscopes. The largest amount of collected bulk sample was processed from the ‘Marco Calcarenite, near Miami’ site with a combined sorted sediment and fossil weight of ~81 g, followed by ~43 g from ‘Marco Calcarenite, Ochre R.’ (53% relative to ‘Marco Calcarenite, near Miami’), ~76 g from ‘Laurier Limestone beds, Edwards Creek’ (94% relative to ‘Marco Calcarenite, near Miami’), and ~52 g from ‘Near Keld base, Vermilion R.’ (64% relative to ‘Marco Calcarenite, near Miami’).

Previously collected vertebrate fossils from the Favel Formation housed at the CMN, FDM, MM, ROM and RSM, fossils donated to the MM fossil collections since 2020, and fossils collected by surface prospecting in 2020 and 2021 and submitted to the MM collections were added to the dataset of previously known Favel Formation vertebrates of MB and Saskatchewan (Kilmury, 2022; Table 1).

**Table 1:** Previously known macro- and microvertebrate (bold font) species occurrences from the Favel Formation in Manitoba and Saskatchewan (Kilmury, 2022). An asterisk (*) indicates taxa represented by museum specimens collected by surface prospecting in 2020 and 2021.

Class					
Species	Family; Order	Marco Calcarenite	Assiniboine Member	Laurier Limestone	Keld Member
**Actinopterygii**					
***Apsopelix anglicus***	Crossognathidae; Crossognathiformes				2
***Cimolichthys* sp.**	Cimolichthyidae; Aulopiformes				1
***Elopopsis* sp.**	Pachyrhizodontidae; Elopiformes				1
***Enchodus petrosus?***	Enchodontidae; Aulopiformes	24			1
***Enchodus shumardi***	Enchodontidae; Aulopiformes				1
***Enchodus* sp. indet.**	Enchodontidae; Aulopiformes	60			4
***Gillicus arcuatus***	Gillicinae; Ichthyodectiformes		1		
Ichthyodectes ctenodon*	Ichthyodectidae; Ichthyodectiformes	1		1	4
Pachyrhizodus minimus*	Pachyrhizodontidae; Elopiformes				1
*Protosphyraena* sp.	Pachycormidae; Pachycormiformes		1		2
Thryptodus loomisi*	Plethodidae; Tselfatiiformes				2
*Xiphactinus* sp. indet.	Ichthyodectidae; Ichthyodectiformes		1		16
Actinopterygii indet.	Family & order indet.	1	3	22	31
**Chondrichthyes**					
Archaeolamna kopingensis*	Archaeolamnidae; Lamniformes	6			
*Cardabiodon ricki*	Cardabiodontidae; Lamniformes				1
Cretalamna appendiculata*	Cretoxyrhinidae; Lamniformes			1	1
*Cretalamna* sp.	Cretoxyrhinidae; Lamniformes				1
***Cretodus* sp.**	Cretoxyrhinidae; Lamniformes				1
***Cretomanta canadensis***	Mobulidae; Myliobatiformes	2			1
Cretoxyrhina denticulata*	Cretoxyrhinidae; Lamniformes			2	7
***Microscyliorhinus* sp.**	Scyliorhinidae; Carcharhiniformes				1
***Odontaspis saskatchewanensis***	Odontaspididae; Lamniformes				2
***Palaeoanacorax pawpawensis***	Anacoracidae; Lamniformes	11			
Ptychodus marginalis*	Ptychodontidae; Hybodontiformes	1			1
Ptychodus occidentalis*	Ptychodontidae; Hybodontiformes				1
Ptychodus rhombodus*	Ptychodontidae; Hybodontiformes			1	2
***Ptychodus rugosus***	Ptychodontidae; Hybodontiformes				4
***Ptychodus* sp.**	Ptychodontidae; Hybodontiformes				2
***Rhinobatos incertus***	Rhinobatidae; Rajiformes				1
***Roulletia canadensis***	Odontaspididae; Lamniformes	4			
***Scapanorhynchus* aff. *S. raphiodon***	Mitsukurinidae; Lamniformes				2
Squalicorax curvatus*	Anacoracidae; Lamniformes			3	8
Squalicorax falcatus*	Anacoracidae; Lamniformes	7		1	6
***Squalicorax* sp. indet.**	Anacoracidae; Lamniformes				3
***Synodontaspis liliae***	Odontaspididae; Lamniformes				1
***Telodontaspis agassizensis***	?Archaeolamnidae; Lamniformes				1
Isuridae indet.	Isuridae; Lamniformes				1
Lamniformes indet.	Family indet.; Lamniformes				1
Chondrichthyes indet.	Family & order indet.		1		2
					
**Reptilia**					
?*Libonectes morgani*	Elasmosauridae; Plesiosauria	1			
*Polycotylus latipinnis*	Polycotylidae; Plesiosauria		1		
*Terminonaris robusta*	Pholidosauridae; Crocodyliformes				2
*Trinacromerum bentonianum*?	Polycotylidae; Plesiosauria				2
Mosasauridae indet.	Mosasauridae; Squamata			1	1
Pliosauridae gen. et sp. nov.	Pliosauridae; Plesiosauria				1
Polycotylid indet.	Polycotylidae; Plesiosauria		1	1	4
Plesiosauria indet.	Family indet.; Plesiosauria				2
**Aves**					
Aves indet.	Hesperornithidae?; Hesperornithiformes		1?		

Fossil specimen images were captured with Nikon D300 camera and Nikkor 18–200 mm lens for macrovertebrate specimens, and for microvertebrate specimens, a Nikon SMZ18 camera with SHR Plan Apo series objective lenses and images captured and stacked using NIS-Elements Imaging Software.

Biogeographic comparisons between the Favel Formation vertebrate assemblage described herein and time-equivalent marine vertebrate assemblages of other North American localities were accomplished using Sorensen’s Coefficient of Community presence-absence analysis. Presence-absence analyses were conducted on both the genus and species levels.

## Results

### Description of indeterminate taxon Vertebrate A

**Figure 4:**
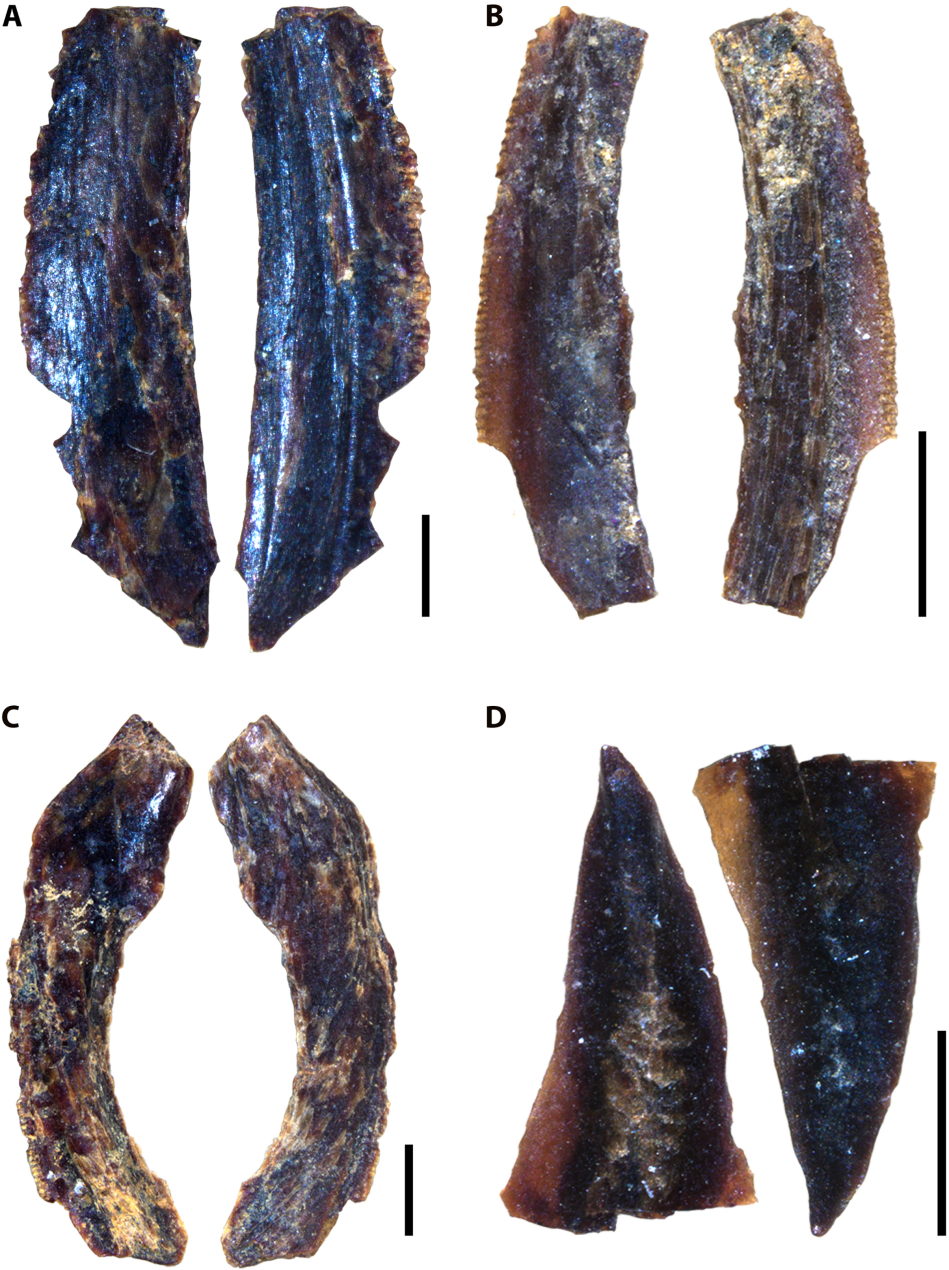
Vertebrate A specimens. (A) Specimen of Vertebrate A (MC Near Miami, MM V-3544). (B) Vertebrate A specimen with well-preserved, serrated edge (MC Ochre R.; MM V-3547). (C) Strongly-curved Vertebrate A specimen (MC Near Miami; MM V-3546). (D) Vertebrate A specimen with preserved distal tip (Laurier Limestone bed; MM V-3548). Orientations: A–D = profile views. Scale: A–D = 1 mm.

Several unknown chondrichthyan and actinopterygian elements were recovered from sampled Favel Formation horizons, including several of one morphotype in particular, referred to here as Vertebrate A (Figs. 4, S1). Fossil elements assigned to Vertebrate A are small (longest fragment 6.78 mm in length), gently to moderately curved with weakly convex lateral faces and wide (0.19–0.76 mm), serrated edges. Most fragments are between 6.39 and 6.78 mm in length, 1.72 and 1.91 mm in maximum width, and 0.54 and 0.57 mm in thickness, with the smallest fragment 1.95 mm long, 1.03 mm wide, and 0.35 mm thick. The width of the thicker middle portion of the element is between 1.06 to 1.28 mm on most specimens and is 0.45 mm wide on the smallest fragment. Serrated edges do not reach the apices and on the distal edges do not reach the base. Each serration along the edges has a tapered point, a height range of 0.11–0.2 mm, a base width range of 0.06–0.16 mm, and where observed, appear to have two layers (Fig. S1). Approximately nine serrations comprise a 1.0 mm distance along the edges. The apex of one specimen (MM V-3548) bears a weakly and distally curved, rounded tip with bladed edges ending approximately 2 mm away from the apex on one edge with incipient serrations, and 1 mm on the other edge, lacking serrations (Fig. 4D).

Vertebrate A elements were nearly all recovered from the mid-Turonian Marco Calcarenite, Favel Formation with one distal end recovered from the second-topmost, 13 cm-thick limestone bed of the early Turonian Laurier Limestone beds, Keld Member (Fig. 4D). The occurrences of nearly all Vertebrate A specimens from the horizon interpreted as nearest to shore, the Marco Calcarenite, and none from the horizon interpreted as furthest from shore, 1 m above the Keld Member base, suggests this species was likely restricted to nearshore environments.

### Faunal assemblage comparisons

Specimens representing previously unknown occurrences recovered from all three bulk-sampled microvertebrate fossil horizons increase the total known apparent diversity of Favel Formation microvertebrate taxa by 14 species, from 26 to 40, and the combined total of macro- and microvertebrate taxa from 42 to 56 species (Tables 1 and 2; Material S1). Total numbers of vertebrate taxa identifiable to order recovered from the three Favel Formation horizons consist of, from most to least: 25 from the Marco Calcarenite, 10 from the Laurier Limestone bed, and 8 from 1 m above Keld base (Table 2). Of the recovered microvertebrate fossil remains from each of the lower, middle, and upper horizons, the most and largest remains were recovered from the upper horizon and the least and smallest from the lower horizon. Preservation of fossil remains is moderate to good from all sites except for remains recovered from the ‘Marco Calcarenite, near Miami’ site, which have poor to moderate preservation because of longer periods of subaerial and subaqueous exposure at a field drainage site.

**Table 2:** Microvertebrate taxa and their abundances of the four sampled Favel Formation sites: MC NM) ‘Marco Calcarenite, near Miami’, MC OR) Marco Calcarenite, Ochre R.’, LLS EC) ‘Laurier Limestone bed, Edwards Creek’, and KB VR) ‘Near Keld base, Vermilion R.’. Taxonomic names in bold represent new occurrences from the Favel Formation of MB.

Class						
Species	Family; Order	MC NM	MC OR	LLS EC	KB VR	Fig.
Actinopterygii						
*Apsopelix* cf. *anglicus*	Crossognathidae; Crossognathiformes	13	6	18	1	Figs. 5J, 7E–7G, 12F–12G
*Apateodus* sp.	Ichthyotringidae; Aulopiformes	2		1	2	Figs. 5A, 9G
*Enchodus petrosus*	Enchodontidae; Aulopiformes			1		Figs. 6B, 9E
*Enchodus* sp.	Enchodontidae; Aulopiformes	6				
*Ichthyodectes ctenodon*	Ichthyodectidae; Ichthyodectiformes	6		8		
***Micropycnodon kansasensis***	Pycnodontidae; Pycnodontiformes	1			1	Figs. 5E, 9H, 10E, 10G, 10I
*Pachyrhizodus minimus*	Pachyrhizodontidae; Elopiformes	2		1		Figs. 6A, 10A
*Protosphyraena* sp.	Pachycormidae; Pachycormiformes	3	1		1	Figs. 9C, 9F
*Xiphactinus* sp.	Ichthyodectidae; Ichthyodectiformes	2	1			Figs. 9A, 9D
**Species A**	Family & order indet.	6				Fig. 11D
**Caturidae indet.**	Caturidae; Amiiformes				1	Fig. 5C
**Pycnodontiformes indet.**	Family indet.; Pycnodontiformes	4				Figs. 10E, 10G, 10I, 10J
**cf. Albulidae *incertae sedis***	Albulidae; Albuliformes	1		1		Fig. 6C
**cf. Plethodidae**	Plethodidae; Tselfatiiformes				1	Fig. 5B
**Teleost A**	Family & order indet.	12	4	1	1	Figs. 5F, 11B, 11E, 11F, 12A
**Teleost B**	Family & order indet.	21				Figs. 10B, 11A, 11K, 12B
**Teleost C**	Family & order indet.	7	3	1		Figs. 10C, 11C
**Teleost D**	Family & order indet.	6	2			Fig. 11L
Actinopterygii indet.	Family & order indet.	>562	>208	>222	>104	
Chondrichthyes						
*Cretomanta canadensis*	Mobulidae; Myliobatiformes	1				
***Ischyrhiza* cf. *I. mira***	Sclerorhynchidae; Rajiformes	1				Fig. 13G
***Ischyrhiza texana***	Sclerorhynchidae; Rajiformes	3				Fig. 13F
*Palaeoanacorax pawpawensis*	Anacoracidae; Lamniformes	1				Fig. 13A
*Rhinobatos incertus*	Rhinobatidae; Rajiformes	3				Figs. 13C, 13D
*Squalicorax curvatus*	Anacoracidae; Lamniformes	2			1	
*Squalicorax* sp. indet.	Anacoracidae; Lamniformes	2				
Chondrichthyes indet.	Family & order indet.	5		4		
Aves						
**cf. *Ichthyornis* sp.**	Ichthyornithidae; Ichthyornithiformes	30	4	6	1	Figs. 5I, 8C, 8D, 14F–14I
Reptilia						
Squamata indet.	Family & order indet.	1				Fig. 4E
**Testudines indet.**	Family & order indet.	7	7	4		Figs. 8A, 8B, 14A–14D
Class indet.	Family & order indet.	>63	>65	>52	>49	
**Vertebrate A**	Family & order indet.	7	1	1		Fig. 4

**Figure 5:**
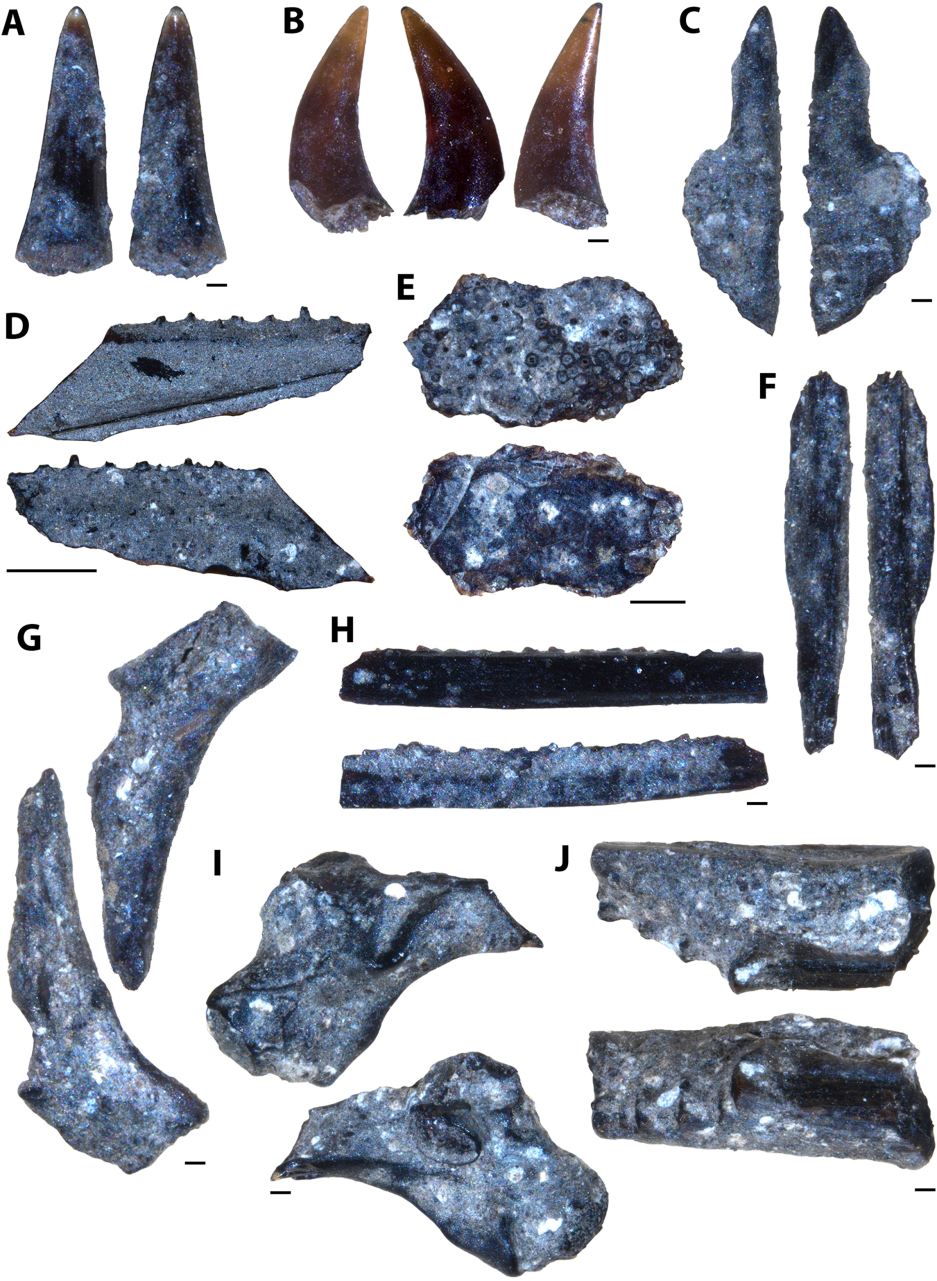
Actinopterygian skull and caudal fin and avian elements collected from 1 m above the Keld Member base, by acid digestion and fossil sorting. (A) *Apateodus* sp. (tooth, MM V-3549). (B) cf. Plethodidae (tooth, MM V-3550). (C) Caturidae indet. (tooth, MM V-3551). (D) sp. indet. (dentary, MM V-3552). (E) *Micropycnodon kansasensis* (prearticular?, MM V-3553). (F) Teleost A (ceratobranchial, MM V-3554). (G) sp. indet. (premaxilla, MM V-3555). (H) sp. indet. (jaw fragment, MM V-3556). (I) cf. *Ichthyornis* sp. (right femur, MM V-3557). (J) *Apsopelix* cf. A. *anglicus* (caudal fin, MM V-3558). Orientations: A, C, left and right = profile views; B, left and middle = profile views, right = lingual view; D, top = lateral view, bottom = mesial view; E, top = occlusal view, bottom = dorsal view; F, left = dorsal view, right = lateral view; G, left = lateral, right = mesial view; H, top and bottom = profile views; I, left = anterior view, right = posterior view; J, top = lateral view, bottom = dorsal or ventral view. Scale: A–C, F–J = 0.1 mm; D = 1 mm; E = 0.5 mm.

#### 1 m above Keld base assemblage

The microvertebrate assemblage recovered from 1 m above Keld base is dominated by remains of bony fishes with at least six species represented (Figs. 5A–5H, 5J). Additionally a single chondrichthyan tooth from *Squalicorax curvatus* and the proximal end of a small cf. *Ichthyornis* sp. femur is reported (Fig. 5I). No reptile remains were recovered (Table 2). The high proportion of singleton species (seven singletons; eight apparent species) from this horizon relative to the middle and upper Favel Formation horizons indicates it has the lowest species coverage (Table 2).

#### Laurier Limestone bed assemblage

**Figure 6:**
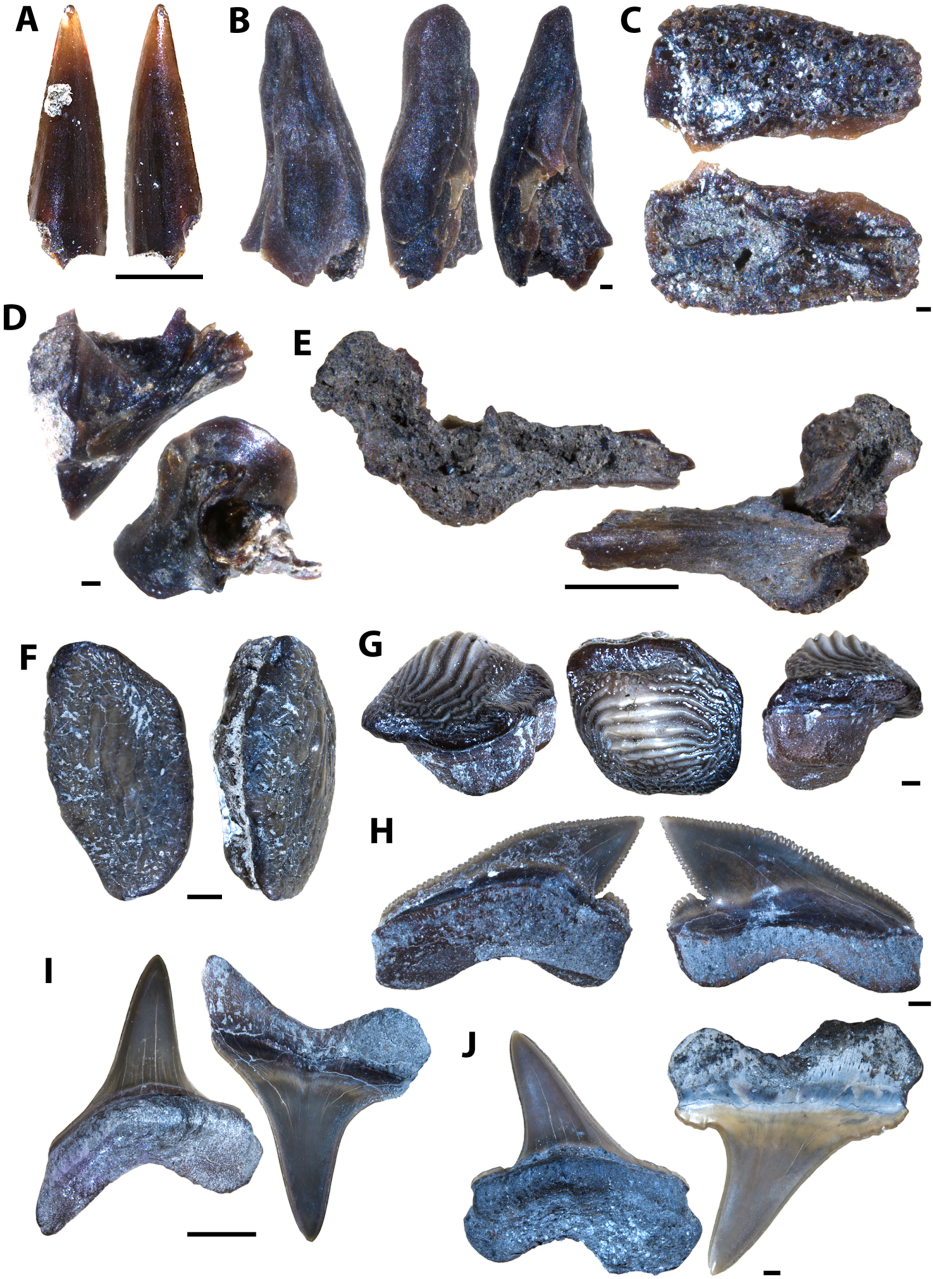
Actinopterygian and chondrichthyan teeth and jaw elements collected from the Laurier Limestone beds, Keld Member, by acid digestion and fossil sorting (A–E), and surface prospecting (F–I). (A) *Pachyrhizodus minimus* (tooth, MM V-3559). (B) *Enchodus petrosus* (palatine tooth, MM V-3560). (C) cf. Albulidae (tooth plate, MM V-3563). (D) sp. undet. (palatine tooth, MM V-3561). (E) sp. undet. (premaxilla, MM V-3562). (F) *Ptychodus rhombodus* (tooth, MM V-3261). (G) *Ptychodus rhombodus* (tooth, MM V-3265). (H) *Squalicorax falcatus* (tooth, MM V-3266). (I) *Cretoxyrhina denticulata* (tooth, MM V-3229). (J) *Cretoxyrhina denticulata* (tooth, MM V-3228). Orientations: A, left and right = profile views; B, left and right = profile views, middle = anterior view; C, top = occlusal view, bottom = dorsal view; D, top = lateral view, bottom = posterior view; E, left = mesial view, right = lateral view; F, left = occlusal view, right = occlusal and profile views; G, left = lingual view, middle = occlusal view, right = labial view; H–J, left = lingual view, right = labial view. Scale: A, E–H, J = 1 mm; B–D = 0.1 mm; I = 5 mm.

**Figure 7:**
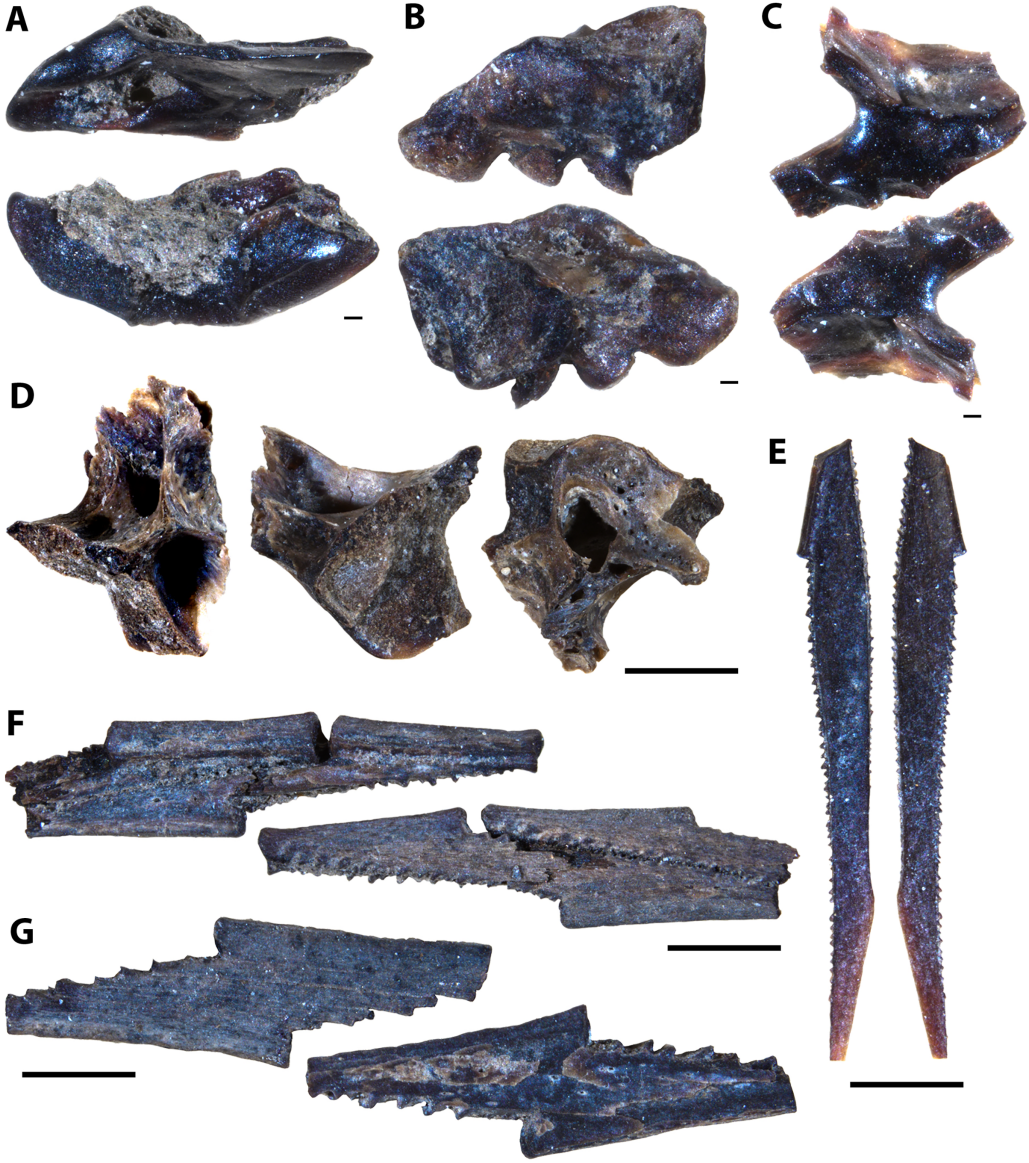
Actinopterygian skull, pectoral, and caudal fin elements collected from the Laurier Limestone beds, Keld Member, by acid digestion and fossil sorting. (A) sp. indet. (prefrontal, MM V-3564). (B) sp. indet. (otolith, MM V-3565). (C) sp. indet. (posttemporal, MM V-3566). (D) sp. indet. (scapula, MM V-3567). (E) *Apsopelix* cf. *A. anglicus* (caudal fin, MM V-3568). (F) *Apsopelix* cf. *A. anglicus* (caudal fin, MM V-3569). (G) *Apsopelix* cf. *A. anglicus* (caudal fin, MM V-3570). Orientations: A, top = ventral view, bottom = dorsal view; B, top = lateral view?, bottom = mesial view?; C, top = mesial view, bottom = lateral view; D, left = posterior view, middle = dorsal view, right = lateral view; E–G, left and right = lateral view. Scale: A–C = 0.1 mm; D–G = 1 mm.

**Figure 8:**
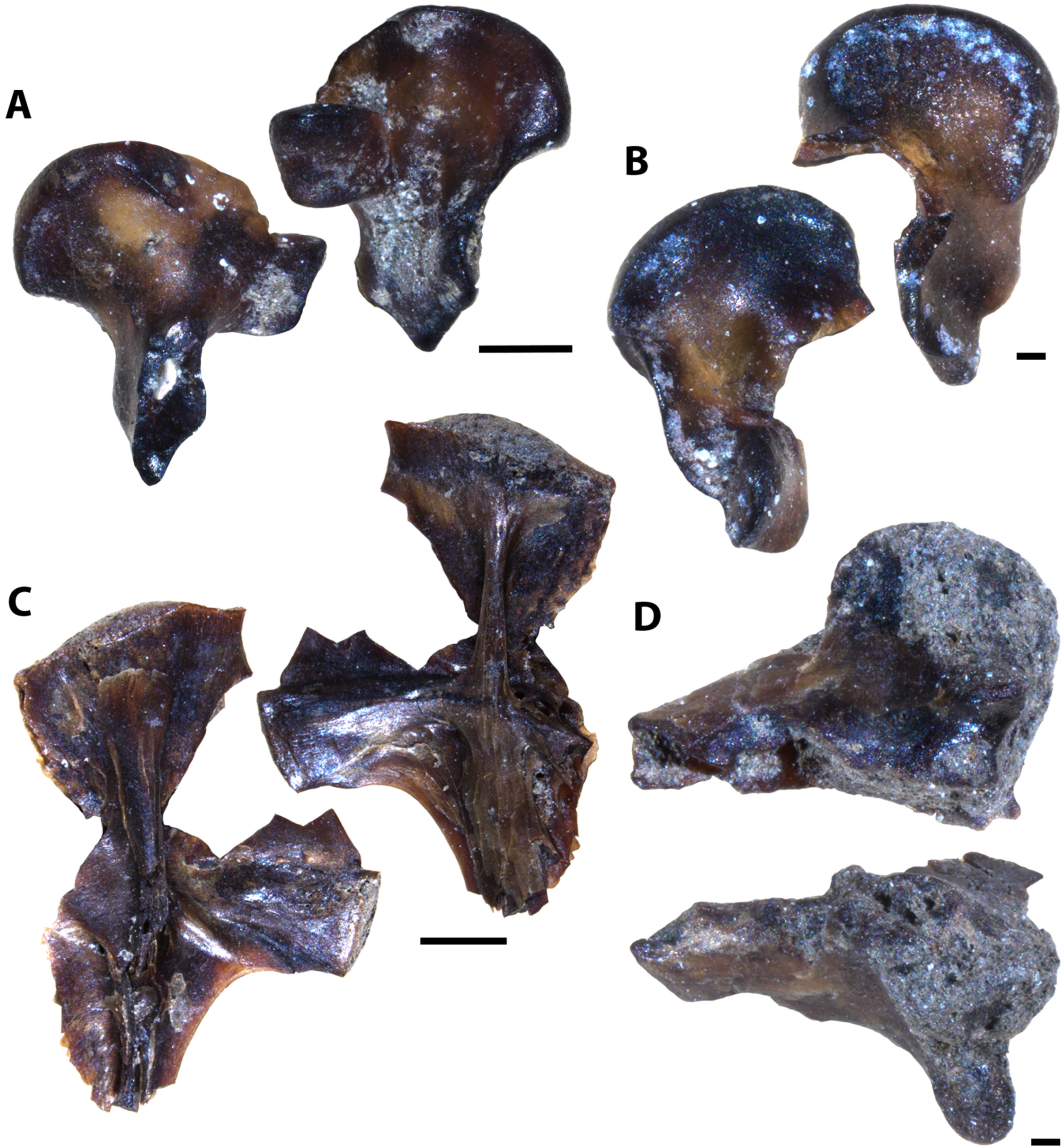
Testudine and avian elements collected from the Laurier Limestone beds, Keld Member, by acid digestion and fossil sorting. (A) Testudines indet. (femur, MM V-3571). (B) Testudines indet. (femur, MM V-3572). (C) cf. *Ichthyornis*(?) sp. (rib, MM V-3573). (D) cf. *Ichthyornis*(?) sp. (femur, MM V-3574). Orientations: A, left = posterior view, right = anterior view; B, left = anterior view, right = posterior view; C, left and right = anterior and posterior views; D, top = posterior view, bottom = anterior view. Scale: A = 0.5 mm; B, D = 0.1 mm; C = 1 mm.

The Laurier Limestone bed assemblage is mostly composed of actinopterygian remains with at least seven species represented (Figs. 6A–6D, 6G, 7). Furthermore a single occurrence of Vertebrate A (Fig. 4G), four partial teeth possibly belonging to rays or skates, four partial limb elements and two ribs belonging to cf. *Ichthyornis* sp. (Figs. 8C and 8D), and four partial femora belonging to an indeterminate testudine are also reported (Figs. 8A and 8B; Table 2). Caudal fin elements referred to cf. *Apsopelix anglicus* (Figs. 7E–7G, S2) are the only species identifiable elements that are more abundant in the Laurier Limestone bed assemblage relative to the lower and upper horizons (Table 2), indicating cf. *A. anglicus* was a common vertebrate community member along the northeastern WIS margin during early Turonian time.

#### Marco Calcarenite assemblage

**Figure 9:**
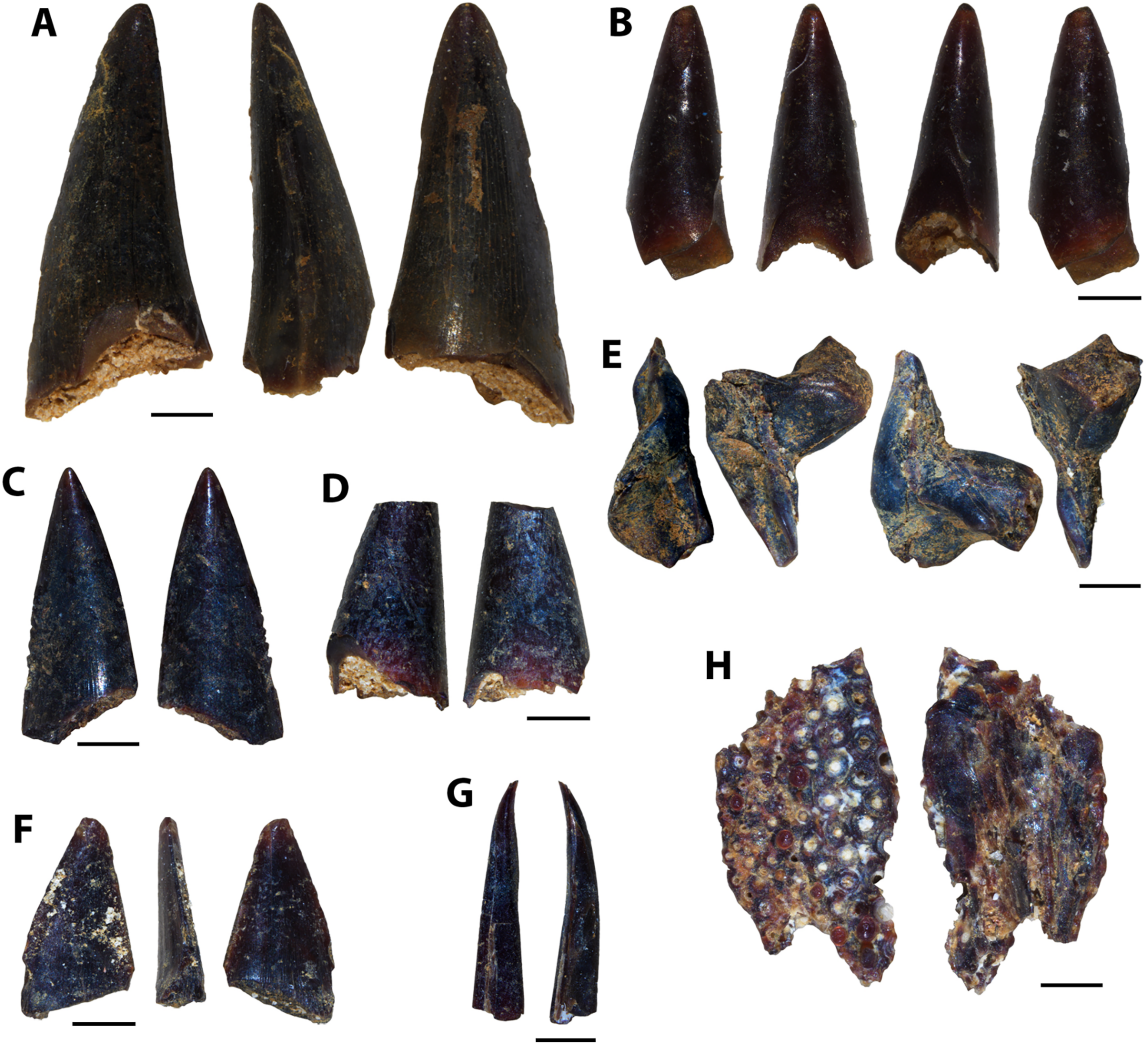
Actinopterygian teeth collected from the Marco Calcarenite, Assiniboine Member, by acid digestion and fossil sorting. (A) *Xiphactinus* sp. (tooth, MM V-3575). (B) sp. indet. (tooth, MM V-3576). (C) *Protosphyraena* sp. (tooth, MM V-3577). (D) *Xiphactinus* sp. (tooth, MM V-3578). (E) *Enchodus petrosus* (palatine tooth and bone, MM V-3579). (F) *Protosphyraena* sp. (tooth, MM V-3580). (G) *?Apateodus* sp. (tooth, MM V-3581). (H) *Micropycnodon kansasensis* (prearticular, MM V-3582). Orientations: A, F, left = lingual view, middle = profile view, right = labial view; B, left and right = profile view, center-left = lingual view, center-right = labial view; C–D, G, left and right = profile view; E, left = anterior view, center-left and center-right = profile view, right = posterior view; H, left = occlusal view, right = dorsal view. Scale: A, C–G = 1 mm; B, H = 0.5 mm.

**Figure 10:**
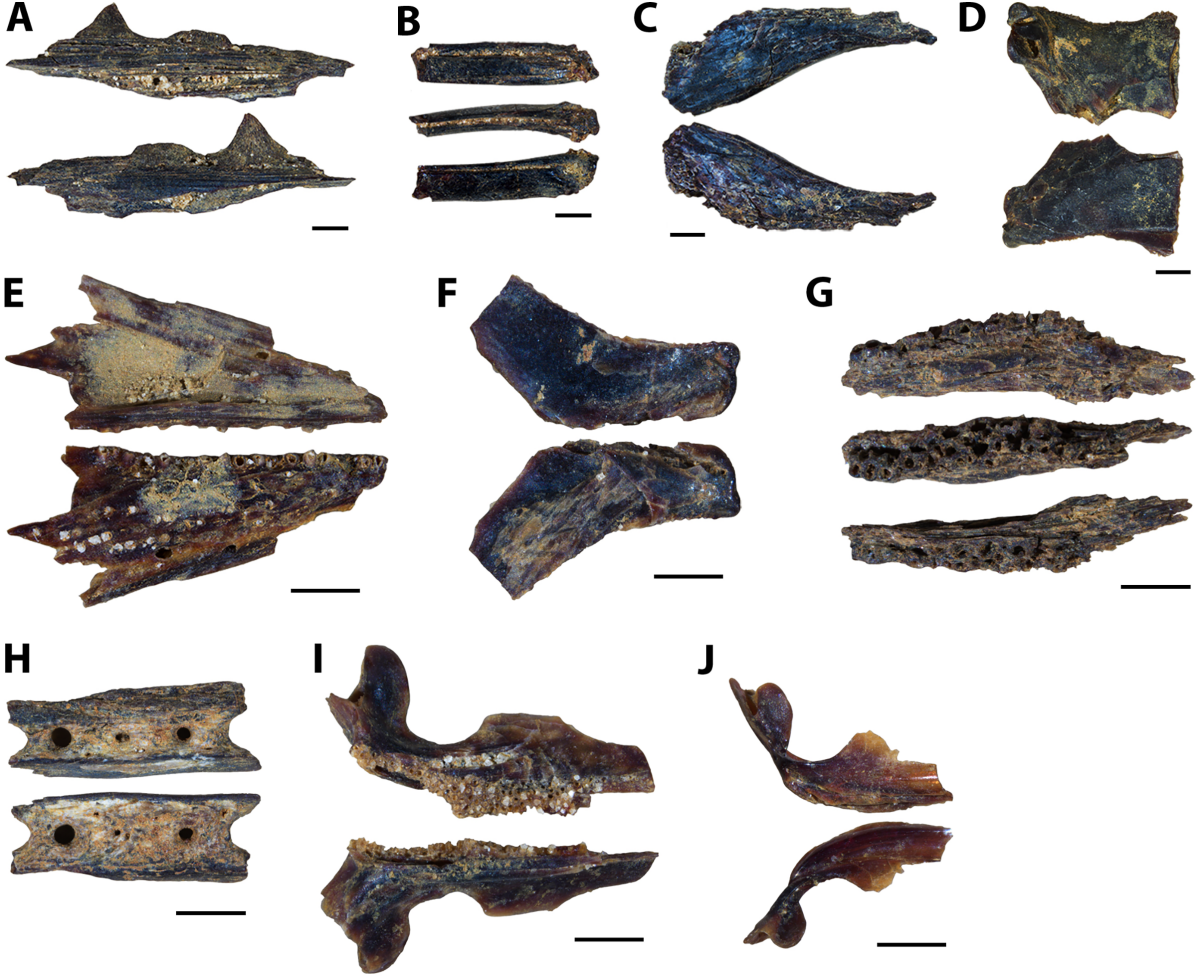
Actinopterygian jaw elements collected from the Marco Calcarenite, Assiniboine Member, by acid digestion and fossil sorting. (A) *Pachyrhizodus* cf. *P. minimus* (jaw fragment, MM V-3583). (B) Teleost B (maxilla, MM V-3584). (C) Teleost C (maxilla, MM V-3585). (D) sp. indet. (maxilla, MM V-3586). (E) ?Pycnodontiformes undet. (dentary, MM V-3587). (F) sp. undet. (maxilla, MM V-3588). (G) ?Pycnodontiformes undet. (tooth plate, MM V-3589). (H) sp. undet. (dentary, MM, V-3590). (I) ?Pycnodontiformes undet. (premaxilla, MM V-3591). (J) ?Pycnodontiformes undet. (premaxilla, MM, V-3592). Orientations: A, top = lateral view, bottom = mesial view; B, top = ventral view, middle = mesial view, bottom = dorsal view; C–D, top = mesial view, bottom = lateral view; E, top = lateral view, bottom = occlusal and mesial views; F, top = lateral view, bottom = mesial view; G, top = lateral view, middle = occlusal view, bottom = occlusal and mesial views; H, top = occlusal view, bottom = ventral view; I–J, top = mesial view, bottom = lateral view. Scale: A–D = 1 mm; E–J = 1 mm.

**Figure 11:**
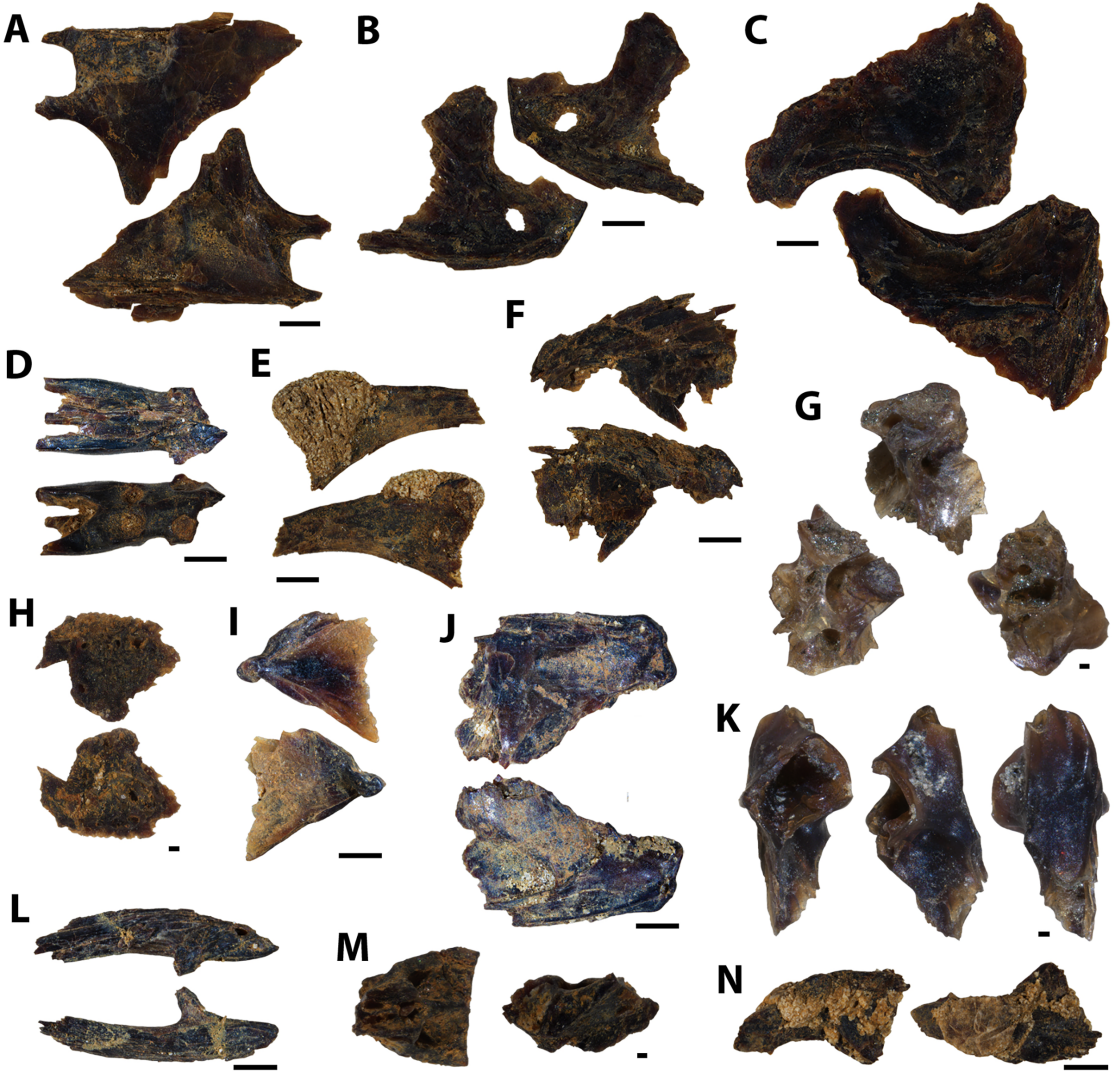
Actinopterygian skull elements collected from the Marco Calcarenite, Assiniboine Member, by acid digestion and fossil sorting. (A) Teleost B (frontal, MM V-3593). (B) Teleost A (left frontal, MM V-3594). (C) Teleost C (frontal, MM V-3595). (D) Species A (frontal, MM V-3596). (E) Teleost A (right frontal, MM V-3597). (F) Teleost A (right frontal, MM V-3598). (G) sp. indet. (prootic, MM V-3599). (H) sp. indet. (sphenotic, MM V-3600). (I) sp. indet. (quadrate, MM V-3601). (J) sp. indet. (prefrontal, MM V-3602). (K) Teleost B (mesethmoid, MM V-3603). (L) Teleost D (posttemporal, MM V-3604). (M) sp. indet. (basioccipital, MM V-3605). (N) sp. indet. (symplectic, MM V-3606). Orientations: A, F, top = dorsal view, bottom = ventral view; B, left = dorsal view, right = ventral view; C–E, J, top = ventral view, bottom = dorsal view; G, left = mesial view, middle = ventral view, right = dorsal view; H, top = lateral view?, bottom = mesial view?; I, top = mesial view, bottom = lateral view; K, left = ventral view, middle = lateral view, right = dorsal view; L, top = lateral view, bottom = mesial view; M, left = ventral view, right = posterior view; N, left = lateral view?, right = mesial view?. Scale: A–F, I–J, L, N = 1 mm; G–H, K, M = 0.1 mm.

**Figure 12:**
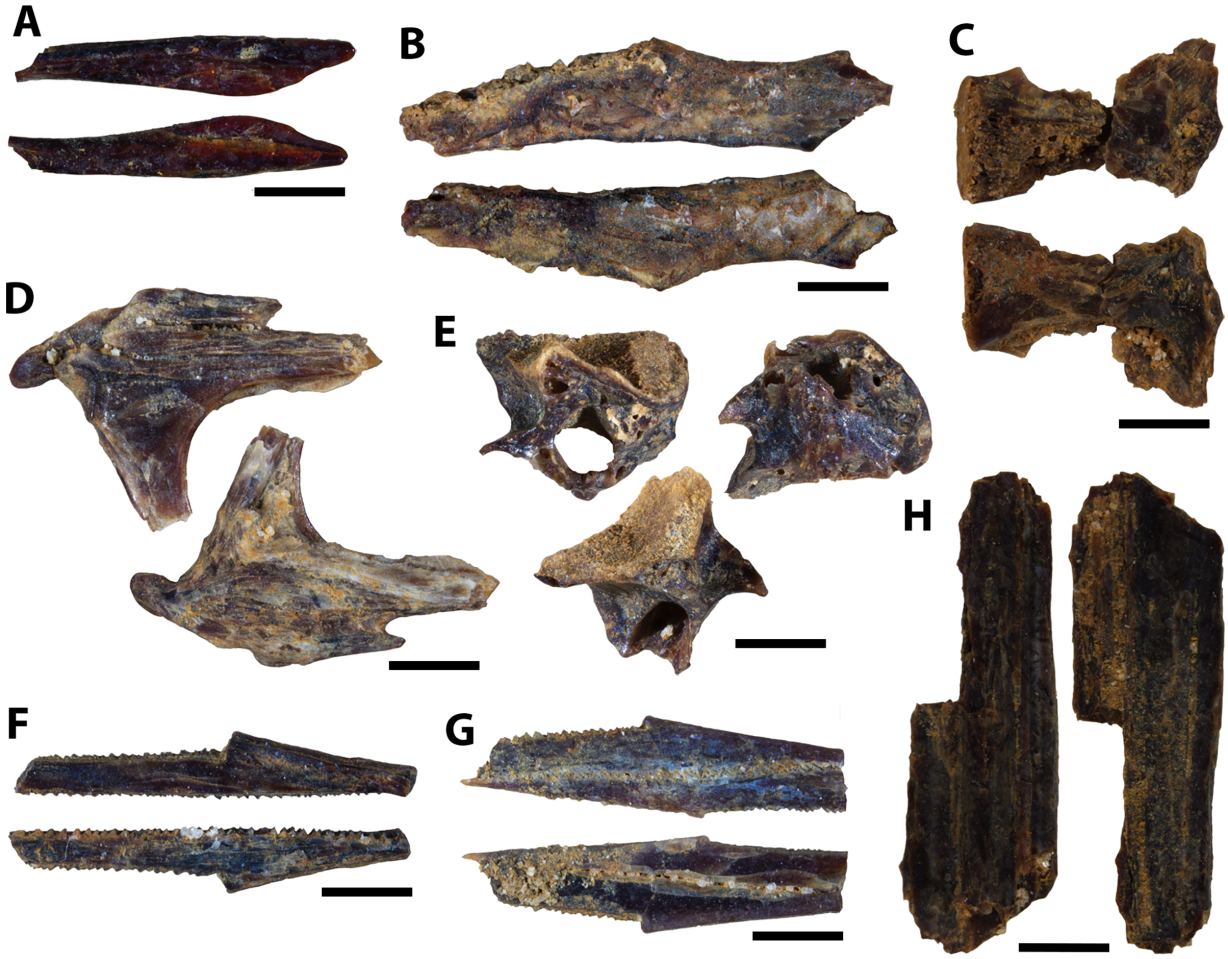
Actinopterygian hyoid arch, pectoral girdle, and caudal fin elements collected from the Marco Calcarenite, Assiniboine Member, by acid digestion and fossil sorting. (A) Teleost A (ceratobranchial, MM V-3607). (B) Teleost B (ceratobranchial, MM V-3608). (C) sp. indet. (radial, MM V-3609). (D) sp. indet. (coracoid, MM V-3610). (E) sp. indet. (scapula, MM V-3611). (F) *Apsopelix* cf. *A. anglicus* (caudal fin, MM V-3612). (G) *Apsopelix* cf. *A. anglicus* (caudal fin, MM V-3613). (H) sp. indet. (epural, MM V-3614). Orientations: A, top = lateral view, bottom = mesial view; B–D, top = mesial view, bottom = lateral view; E, left = mesial view, middle = lateral view, right = anterior view; F–G, top and bottom = lateral view; H, left and right = lateral view. Scale: A–H = 1 mm.

**Figure 13:**
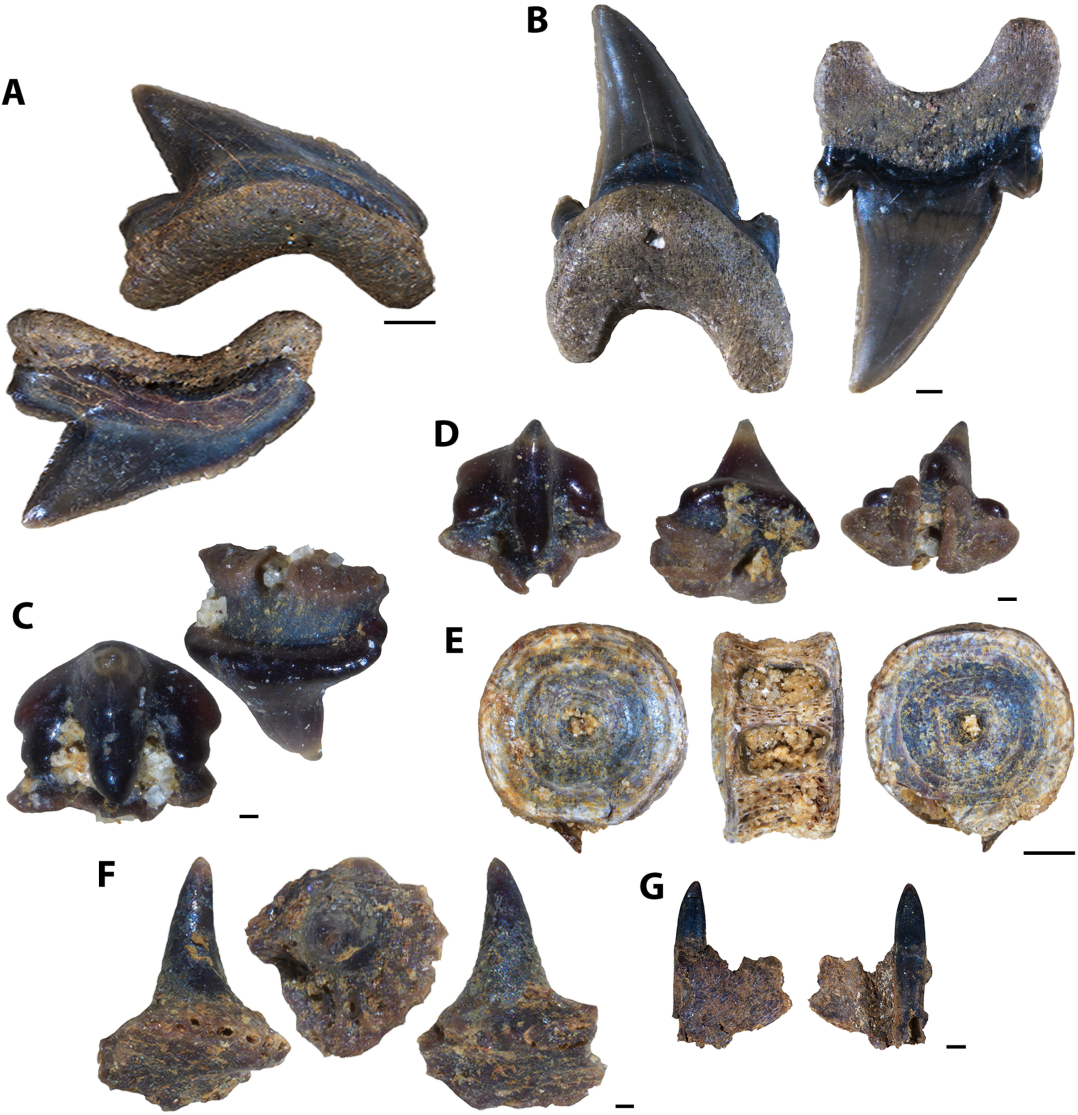
Chondrichthyan elements collected from the Marco Calcarenite, Assiniboine Member, by surface prospecting and by acid digestion and fossil sorting. (A) *Palaeoanacorax pawpawensis* (tooth, MM V-3615). (B) *Archaeolamna* ex gr. *kopingensis* (tooth, MM V-3219). (C) *Rhinobatos incertus* (tooth MM V-3270). (D) *Rhinobatos incertus* (tooth MM V-3270). (E) sp. indet. (vertebra, MM V-). (F) *Ischyrhiza texana* (rostral tooth, MM V-3617). (G) *Ischyrhiza* cf. *I. mira* (rostral tooth, MM V-3618). Orientations: A, top = lingual view, bottom = labial view; B–C, left = lingual view, right = labial view; D, left = lingual view, middle = distal and labial views, right = lingual and basal views; E, left and right = articular views, middle = ventral view; F, left = dorsal view, middle = apical view, right = posterior view; G, left = dorsal view, right = ventral view. Scale: A–B, E, G = 1 mm, C–D, F = 0.1 mm.

**Figure 14:**
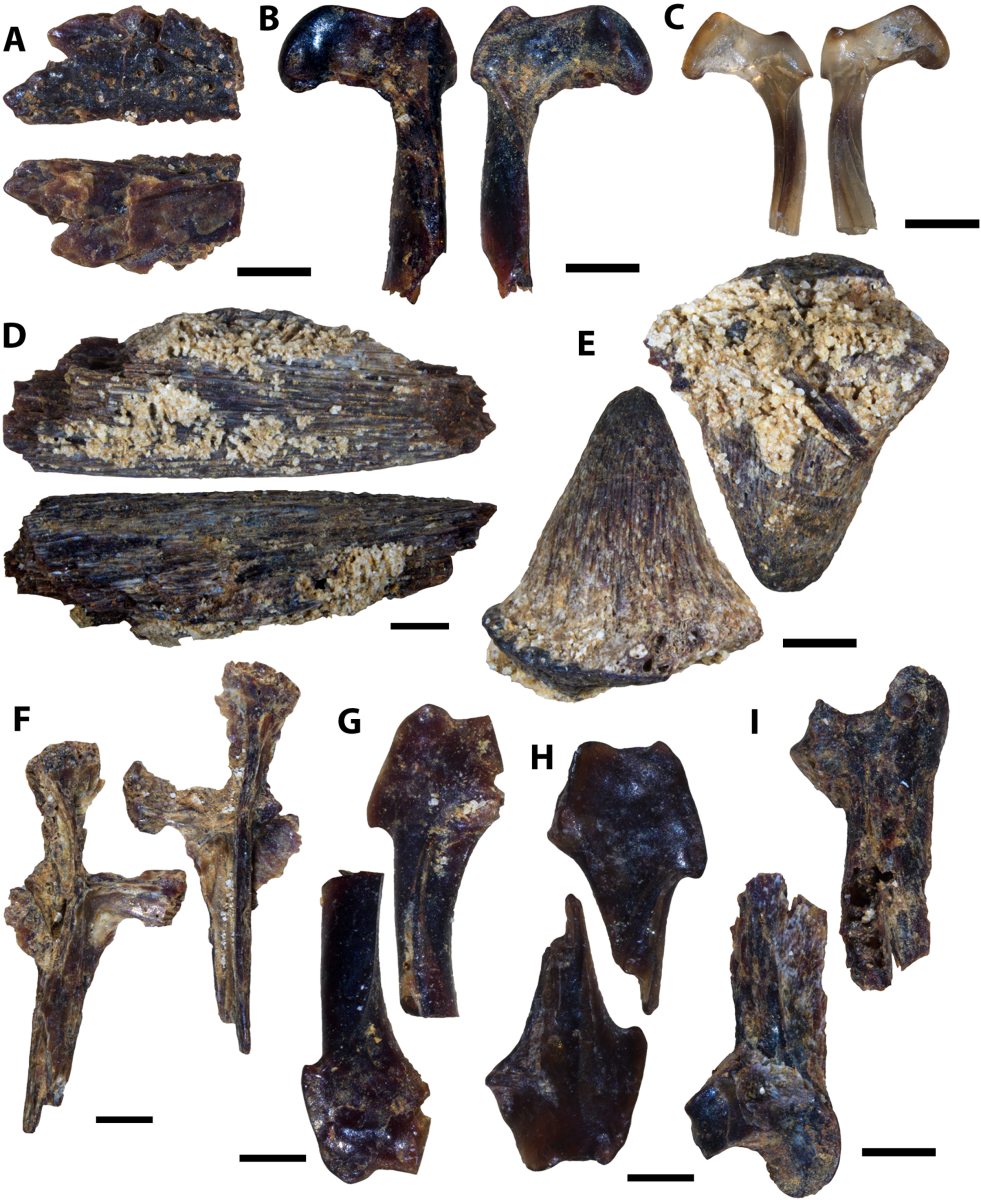
Testudine, squamate, and avian elements collected from the Marco Calcarenite, Assiniboine Member, by acid digestion and fossil sorting. (A) Testudines indet. (carapace fragment, MM V-3619). (B) Testudines indet. (left femur, MM V-3620). (C) Testudines indet. (right femur, MM V-3621). (D) Testudines indet. (rib, MM V-3622). (E) Squamata indet, (tooth, MM V-3623). (F) cf. *Ichthyornis*(?) sp. (rib, MM V-3624). (G) cf. *Ichthyornis* sp. (humerus, MM V-3625). (H) cf. *Ichthyornis* sp. (humerus, MM V-3626). (I) cf. *Ichthyornis* sp. (femur, MM V-3627). Orientations: A, top = dorsal view, bottom = ventral view; B, left = anterior view, right = posterior view; C, left = posterior view, right = anterior view; D, top and bottom = sides indet.; E, left and right = profile views; F, left and right = anterior and posterior views; G–H, left = lateral view, right = mesial view; I, left = anterior view, right = posterior view. Scale: A–F, I = 1 mm; G–H = 0.5 mm.

The microvertebrate assemblage sampled from the ‘Marco Calcarenite, near Miami’ site contains the highest abundance of fossil elements, mostly comprised of actinopterygian vertebrae, and greatest diversity of represented taxa with at least 14 actinopterygian, nine chondrichthyan, one avian, one squamate, and one testudine species represented (Figs. 9–14; Table 2).

The ‘Marco Calcarenite, Ochre R.’ microvertebrate assemblage contains actinopterygian, chondrichthyan, avian, and reptilian occurrences that are all also represented from the ‘Marco Calcarenite, near Miami’ assemblage and is comparatively less diverse, most likely because the amount of sorted sediment and fossils is about half (53%) of that sorted from ‘Marco Calcarenite, near Miami’ and not representative of a truly less diverse assemblage. Interestingly, no ray or skate remains were recognized from the ‘Marco Calcarenite, Ochre R.’ assemblage (Table 2) which may indicate that as with the lower apparent diversity, is a result of less sampling coverage through sediment and fossil sorting and the lower probability of recovering less common microvertebrate species.

### Preservational biases

**Figure 15:**
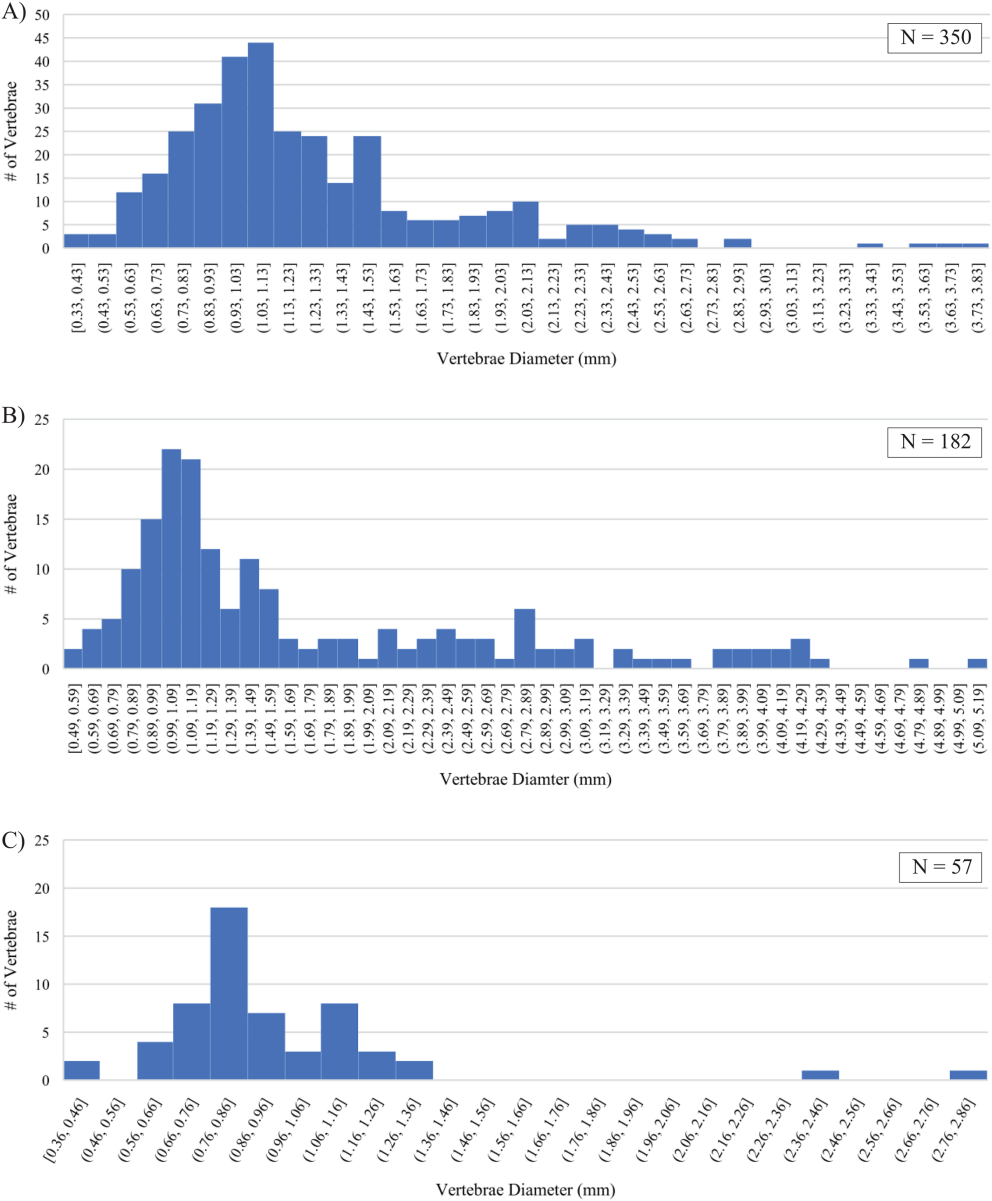
Size distributions of actinopterygian vertebrae diameters. (A) ‘Marco Calcarenite, Ochre R.’. (B) ‘Marco Calcarenite, near Miami’. (C) ‘Laurier Limestone beds, Edwards Creek’.

Differences in fossil preservation, most represented size classes, and their size distributions preserved within the three sampled horizons are recognized. Strong similarities between the Marco Calcarenite assemblages from both sampled sites and differences between them and the Laurier Limestone bed assemblage are apparent in measurements of actinopterygian vertebrae (Fig. 15; Material S2). The best preserved and smallest fossils were recovered from the lowest and middle horizons, 1 m above the Keld Member base along Vermilion R. and the second topmost Laurier Limestone bed along Edwards Creek, respectively. The Laurier Limestone bed assemblage vertebrae size distribution ranges between 0.36 and 2.82 mm in diameter and contains two peaks with the most vertebrae (17) in the 0.76–0.86 mm size class and the second-most vertebrae (8) in the 1.06–1.16 mm size class (Fig. 15C). The low number (21) and poor preservation of vertebrae recovered from the ‘Near Keld base, Vermilion R.’ site did not allow for reliable assessment of vertebrae size distribution from the lowest horizon. Although not as well preserved, the Marco Calcarenite horizon at the ‘Marco Calcarenite, near Miami’ site yielded the most and the largest microvertebrate remains, with a size distribution range of 0.49–5.14 mm and the 0.99–1.09 and 1.09–1.19 mm size classes containing the most vertebrae (22 and 21, respectively; Fig. 15B). The highest number of vertebrae (350) were measured from the ‘Marco Calcarenite, Ochre R.’ site and the size classes with the most vertebrae are nearly identical to those of the ‘Marco Calcarenite, near Miami’ site, with 41 in the 0.93–1.03 mm size class and 44 in the 1.03–1.13 mm size class, though its 0.33–3.81 mm vertebrae size distribution range is more similar to that of the ‘Laurier Limestone beds, Edwards Creek’ site than the ‘Marco Calcarenite, near Miami’ site (Figs. 15A–15C). Similar size distributions between the two sites with the least and most measured vertebrae, ‘Laurier Limestone beds, Edwards Creek’ and ‘Marco Calcarenite, Ochre R.’, respectively, suggest their microvertebrate assemblages were preserved under similar, low-energy conditions, with the large difference in amounts of recovered vertebrae indicating the sampled horizon at ‘Marco Calcarenite, Ochre R.’ as closer to shore relative to the sampled Laurier Limestone bed where both higher sedimentation rates and greater abundances of small-bodied actinopterygians allowed for preservation of more vertebrae. The relatively low number of recovered vertebrae and noticeable gap of measured vertebrae in the 1.36–2.36 mm size classes of the Laurier Limestone bed assemblage (Fig. 15C) may represent lower fossil preservation potential, particularly of relatively large vertebrae, in a depositional environment with a lower sedimentation rate relative to that of the Marco Calcarenite. The larger fossil elements represented in the ‘Marco Calcarenite, near Miami’ and greater vertebrae size distribution range of 0.49–5.14 mm indicates a depositional environment with higher sedimentation rates capable of rapidly burying and preserving larger fossil elements relative to the those of the ‘Laurier Limestone beds, Edwards Creek’ and ‘Marco Calcarenite, Ochre R.’ sampled horizons. The apparent absence of vertebrae with measured diameters less than 0.49 mm, shape of vertebrae size distribution (Fig. 15B), and poor to moderate preservation of most fossil elements suggests the horizon sampled at ‘Marco Calcarenite, near Miami’ experienced some degree of sediment reworking with preferential preservation bias of fossil elements larger than 0.8–1.0 mm and also supports the interpretation of this horizon as being deposited under high-energy conditions.

### Paleoenvironmental interpretations

Clay to fine-sized sediment, the absence of quartz grains, and the low number of recovered vertebrae (21) indicates the ‘Near Keld base, Vermilion R.’ site as furthest offshore from the eastern WIS shoreline relative to the middle and upper Favel Formation horizons. The absence of several taxa associated with nearshore marine habitats, including *Ischyrhiza mira*, *I. texana*, and *Rhinobatos incertus* (Welton & Farish, 1993; Everhart, 2007), also supports ‘Near Keld base, Vermilion R.’ as the site furthest from the WIS shoreline. The exceptional preservation of fossil elements, clay to fine sediment grain sizes, and high abundance of small fossil elements recovered from the Laurier Limestone bed horizon relative to the sampled lower and upper Favel Formation horizons suggests a low energy depositional environment closer to shore than the lower horizon and further from shore than the upper horizon. The relatively poorly sorted sediment and fossil size classes, high abundance of quartz grains and inoceramid clam fragments, and presence of several taxa associated with nearshore environments, including the actinopterygian cf. Albulidae *incertae sedis* (Cumbaa, Underwood & Schröder-Adams, 2013) and chondrichthyans *I. mira*, *I. texana*, and *R. incertus* (Welton & Farish, 1993; Everhart, 2007), suggests the Marco Calcarenite represents the highest energy and nearest to shore depositional environment relative to the lower and middle Favel Formation horizons, with the ‘Marco Calcarenite, near Miami’ site situated closer to shore than the ‘Marco Calcarenite, Ochre R.’ site.

## Discussion

### Favel Formation marine cycle trends

Faunal analyses of microvertebrate assemblages from the Favel Formation of MB has substantially improved understanding of vertebrate communities from the northeastern WIS margin by increasing representation of small-bodied vertebrates. Relative proximity to shore is estimated for all horizons from the four sampled sites with reference to the size range and size class distribution of actinopterygian vertebrae, sediment grain size range, and additionally supported by the presence or absence of vertebrate taxa associated with nearshore habitats. Relative positions from the WIS northeastern shoreline for the four analyzed microvertebrate assemblages are as follows, in order of stratigraphically lowest to highest: ‘Near Keld base, Vermilion R.’ as furthest offshore, ‘Laurier Limestone beds, Edwards Creek’ as offshore, ‘Marco Calcarenite, Ochre R.’ as nearshore, and ‘Marco Calcarenite, near Miami’ as nearest to shore. The overall regression from early to mid-Turonian time indicated by the Favel Formation microvertebrate assemblages of MB is consistent with other interpretations of WIS sea level change during this time interval (*e.g*., Schröder-Adams, 2014) and represents the overall regressive phase of the Greenhorn WIS third-order marine cycle.

### Faunal occurrences

The planktonic foraminiferal species *Heterohelix globulosa* was observed within the 0.42–0.83 mm size class of sediment sorted from the ‘Near Keld base, Vermilion R.’ horizon (Fig. S3). The presence of *H. globulosa* further supports the stratigraphic position of the ‘Near Keld base, Vermilion R.’ horizon as within the Keld Member, though does not distinguish whether the horizon is of late Cenomanian or early Turonian age because of its chronostratigraphic range (McNeil & Caldwell, 1981; Dionne, Schröder-Adams & Cumbaa, 2016). Although examination was brief, the presence of *H. globulosa* and apparent absence of *Clavihedbergella simplex* and *Whiteinella aprica* indicates the ‘Near Keld base, Vermilion R.’ horizon is of late Cenomanian age (Dionne, Schröder-Adams & Cumbaa, 2016).

Teeth of benthic chondrichthyan species not previously recognized from the mid-Turonian strata of MB and associated with nearshore habitats (Welton & Farish, 1993) were recovered from the ‘Marco Calcarenite, near Miami’ site, including the sawfishes *Ischyrhiza mira* and *I. texana* (Tables 2 and 3), though none were recognized from the more offshore, lowest horizon at the ‘Near Keld base, Vermilion R.’ site. Recognition of the Marco Calcarenite as representing a high-energy, nearshore depositional environment based upon the presence of taxa associated with nearshore habitats, relatively high abundance of quartz grains and inoceramid fragments, and poorly sorted sediment and fossil size classes agrees with the mid-Turonian timing of the Greenhorn marine cycle regressive phases (Schröder-Adams, 2014; Bamburak, Hamilton & Heaman, 2016) and is significant since it provides further insight into the enigmatic position of the northeastern WIS shoreline.

**Table 3:** Summary list of all currently known macro- and microvertebrate (bold font) species occurrences from the Favel Formation in Manitoba and Saskatchewan (Kilmury, 2022).

Class					
Species	Family; Order	Marco Calcarenite	Assiniboine Member	Laurier Limestone	Keld Member
**Actinopterygii**					
***Apsopelix anglicus***	Crossognathidae; Crossognathiformes	19		18	3
***Apateodus* sp.**	Ichthyotringidae; Aulopiformes	2		1	2
***Cimolichthys* sp.**	Cimolichthyidae; Aulopiformes				1
***Elopopsis* sp.**	Pachyrhizodontidae; Elopiformes				1
***Enchodus petrosus***	Enchodontidae; Aulopiformes	25		1	1
***Enchodus shumardi***	Enchodontidae; Aulopiformes				1
***Enchodus* sp. indet.**	Enchodontidae; Aulopiformes	66			4
***Gillicus arcuatus***	Gillicinae; Ichthyodectiformes		1		
***Ichthyodectes ctenodon***	Ichthyodectidae; Ichthyodectiformes	7		9	4
***Micropycnodon kansasensis***	Pycnodontidae; Pycnodontiformes	4			1
***Pachyrhizodus minimus***	Pachyrhizodontidae; Elopiformes	2		1	1
*Protosphyraena* sp.	Pachycormidae; Pachycormiformes	4	1		3
*Thryptodus loomisi*	Plethodidae; Tselfatiiformes				2
*Xiphactinus* sp. indet.	Ichthyodectidae; Ichthyodectiformes	3	1		16
**Species A**	Family & order indet.	6			
**Caturidae indet.**	Caturidae; Amiiformes				1
**Pycnodontiformes indet.**	Family indet.; Pycnodontiformes	4			
**cf. Albulidae *incertae sedis***	Albulidae; Albuliformes	1		1	
**cf. Plethodidae**	Plethodidae; Tselfatiiformes				1
**Teleost A**	Family & order indet.	16		1	1
**Teleost B**	Family & order indet.	21			
**Teleost C**	Family & order indet.	10		1	
**Teleost D**	Family & order indet.	8			
Actinopterygii indet.	Family & order indet.	>771	3	>244	>135
**Chondrichthyes**					
*Archaeolamna kopingensis*	Archaeolamnidae; Lamniformes	6			
*Cardabiodon ricki*	Cardabiodontidae; Lamniformes				1
*Cretalamna appendiculata*	Cretoxyrhinidae; Lamniformes			1	1
*Cretalamna* sp.	Cretoxyrhinidae; Lamniformes				1
***Cretodus* sp.**	Cretoxyrhinidae; Lamniformes				1
***Cretomanta canadensis***	Mobulidae; Myliobatiformes	3			1
*Cretoxyrhina denticulata*	Cretoxyrhinidae; Lamniformes			2	7
***Ischyrhiza* cf. *I. mira***	Sclerorhynchidae; Rajiformes	1			
***Ischyrhiza texana***	Sclerorhynchidae; Rajiformes	3			
***Microscyliorhinus* sp.**	Scyliorhinidae; Carcharhiniformes				1
***Odontaspis saskatchewanensis***	Odontaspididae; Lamniformes				2
***Palaeoanacorax pawpawensis***	Anacoracidae; Lamniformes	14			
***Ptychodus marginalis***	Ptychodontidae; Hybodontiformes	1			1
***Ptychodus occidentalis***	Ptychodontidae; Hybodontiformes				1
***Ptychodus rhombodus***	Ptychodontidae; Hybodontiformes			1	2
***Ptychodus rugosus***	Ptychodontidae; Hybodontiformes				4
***Ptychodus* sp.**	Ptychodontidae; Hybodontiformes				2
***Rhinobatos incertus***	Rhinobatidae; Rajiformes	3			1
***Roulletia canadensis***	Odontaspididae; Lamniformes	4			
***Scapanorhynchus* aff. *S. raphiodon***	Mitsukurinidae; Lamniformes				2
***Squalicorax curvatus***	Anacoracidae; Lamniformes			3	8
***Squalicorax falcatus***	Anacoracidae; Lamniformes	7		1	6
***Squalicorax* sp. indet.**	Anacoracidae; Lamniformes	2			3
***Synodontaspis liliae***	Odontaspididae; Lamniformes				1
***Telodontaspis agassizensis***	?Archaeolamnidae; Lamniformes				1
Isuridae indet.	Isuridae; Lamniformes				1
Lamniformes indet.	Family indet; Lamniformes				1
Chondrichthyes indet.	Family & order indet.	5	1	4	2
**Reptilia**					
?*Libonectes morgani*	Elasmosauridae; Plesiosauria	1			
*Polycotylus latipinnis*	Polycotylidae; Plesiosauria		1		
*Terminonaris robusta*	Pholidosauridae; Crocodyliformes				2
*Trinacromerum bentonianum*?	Polycotylidae; Plesiosauria				2
Mosasauridae indet.	Mosasauridae; Squamata			1	1
Pliosauridae gen. et sp. nov.	Pliosauridae; Plesiosauria				1
Polycotylid indet.	Polycotylidae; Plesiosauria		1	1	4
Plesiosauria indet.	Family indet.; Plesiosauria				2
Squamata indet.	Family & order indet.	1			
**Testudines indet.**	Family & order indet.	14		4	
**Aves**					
**cf. *Ichthyornis* sp.**	Ichthyornithidae; Ichthyornithiformes	34		6	1
Aves indet.	Hesperornithidae?; Hesperornithiformes		1?		
Class indet.	Family & order indet.	>128		>52	>49
**Vertebrate A**	Family & order indet.	8		1	

Although fully articulated skulls of *Cimolichthys* sp. (Fig. S4) and *Pachyrhizodus minimus* (Fig. S5) have been recovered from the Keld Member, only disarticulated elements corresponding to *P. minimus* were identified from the Marco Calcarenite and Laurier Limestone bed horizons (Fig. 6A, 10A; Table 2).

Several bony fish skull and hyoid arch elements collected from the four sampled sites were recognized as similar to those of modern bony fish taxa. Actinopterygian material assigned to teleost A–D (Figs. 5, 10–12; Tables 2 and 3) share anatomical similarities with the following modern fish taxa: Teleost A with Family Pleuronectidae, Teleost B with Family Gadidae, Teleost C with Family Scorpaenidae, and Teleost D with Salmonidae (Cannon, 1987). Whether the anatomical similarities between the teleost A-D material and modern fish taxa are due to close phylogenetic relationships or are a result of occupying similar ecological niches has yet to be determined.

The indeterminate element, Vertebrate A, may belong to skull, branchial arch, or fin elements of common Turonian fish, such as *A. anglicus*. Remains of Vertebrate A are fragile relative to most recovered microvertebrate fossils due to their small size and thin shape and the majority of occurrences were from the Marco Calcarenite horizon deposited under relatively high-energy conditions, which may explain why complete elements were not observed.

### Biogeographical implications

**Figure 16:**
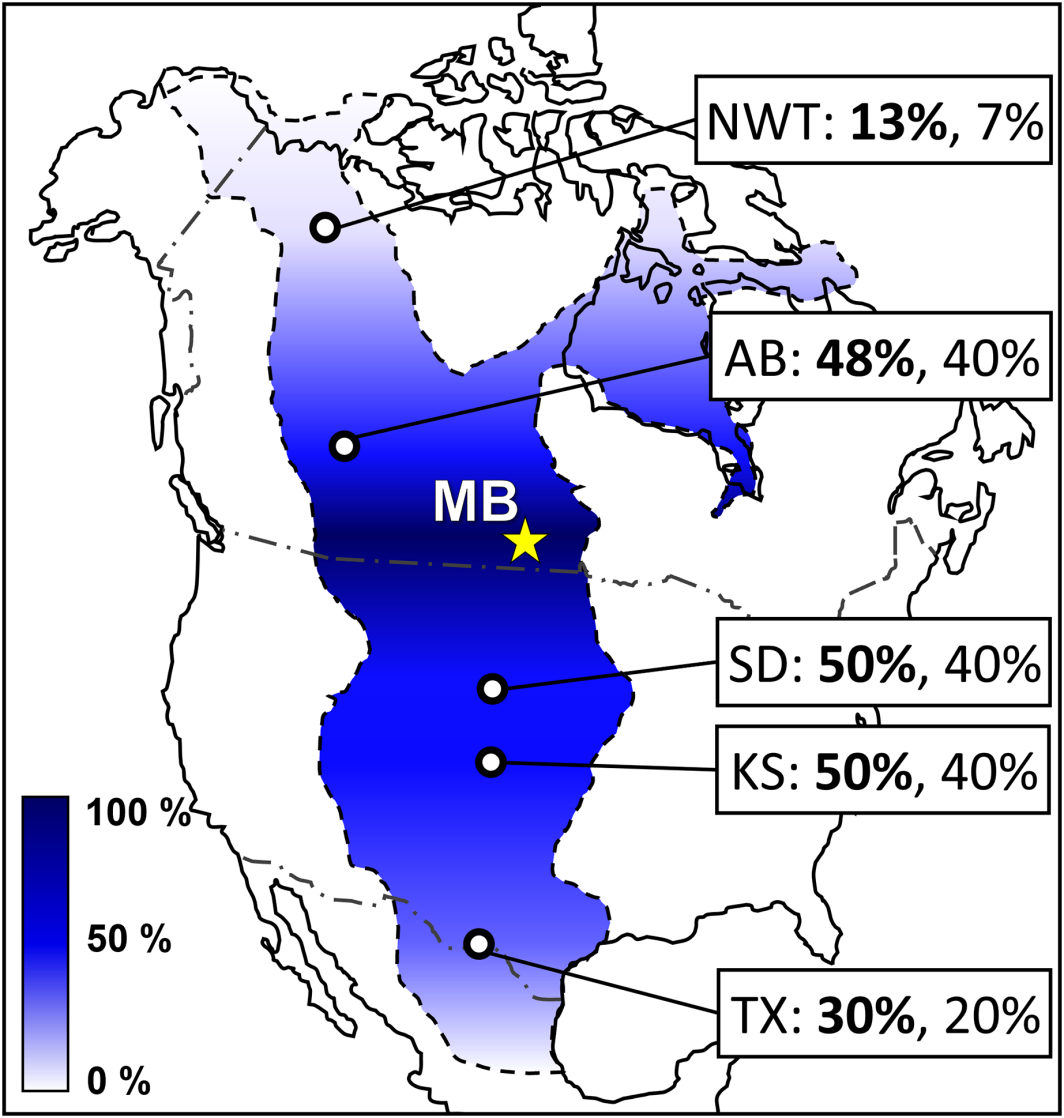
Colorimetric map showing presence-absence vertebrate biogeography of the late Cenomanian to mid-Turonian Western Interior Seaway relative to the MB escarpment. Colorimetric scale is defined using genus-level Coefficient of Community (CC) values (Table S1) relative to the updated MB escarpment Favel Fm vertebrate assemblage (Table 3), with 0% (completely dissimilar) as white and 100% (completely similar) as dark blue. Site labels include CC values at the genus-level (bold) and species-level (normal). Abbreviations and data citations: NWT, Northwest Territories (Cumbaa et al., 2018; Kilmury & Brink, 2022); AB, Watino, Alberta (Fox, 1984; Wilson & Chalifa, 1989; Cook et al., 2013); MB, Manitoba escarpment (Table 3; Kilmury, 2022); SD, South Dakota (Kilmury & Brink, 2022); KS, Kansas (Bice & Shimada, 2016; McIntosh, Shimada & Everhart, 2016; Kilmury & Brink, 2022); and TX, Texas (Kilmury & Brink, 2022).

New occurrences of actinopterygians cf. Albulidae *incertae sedis*, *Micropycnodon kansasensis*, *Pachyrhizodus minimus* (Fig. S5), *Protosphyraena* sp., and *Thryptodus loomisi* (Fig. S6B), chondrichthyans *Ischyrhiza mira*, *I. texana*, *Ptychodus marginalis* (Fig. S6A), *P. occidentalis*, and *P. rhombodus*, the avian cf. *Ichthyornis* sp., and Testudines indet. (Tables 1–3) increases the relatively strong biogeographic affinity between the MB and South Dakota mid-Turonian marine vertebrate assemblages, as well as weaker biogeographic affinities with WIS localities further south in Kansas and Texas (Welton & Farish, 1993; Bice & Shimada, 2016; Kilmury & Brink, 2022). The strengthened biogeographic relationships are demonstrated with the results of a presence-absence analysis (Table S1) shown in Fig. 16 comparing the updated Favel Fm vertebrate faunal assemblage of the MB escarpment (Table 3) with other time-equivalent Western Interior Seaway assemblages in Northwest Territories (Cumbaa et al., 2018; Kilmury & Brink, 2022), west-central Alberta (Fox, 1984; Wilson & Chalifa, 1989; Cook et al., 2013), South Dakota (Kilmury & Brink, 2022), Kansas (Bice & Shimada, 2016; McIntosh, Shimada & Everhart, 2016; Kilmury & Brink, 2022), and Texas (Kilmury & Brink, 2022). The increased biogeographic affinities with WIS localities south of MB provides further support for a relatively large central vertebrate community zone distinct from northern and southern ones during late Cenomanian to early Turonian time (Fig. 16), as well as decreases the gradient of the north-south or central-south community boundary positioned between WIS localities in present-day South Dakota and Kansas during early and mid-Turonian times (Kilmury & Brink, 2022). Since there was only a minor increase in specimens belonging to taxa shared between the MB Keld Member and coeval Northwest Territories Unit E vertebrate assemblages, which include *Cimolichthys* sp., *Enchodus petrosus*, and *Ichthyodectes ctenodon*, nor recognition of taxa specific to the Unit E assemblage (Cumbaa & Murray, 2008; Murray & Cumbaa, 2013, 2015; Murray, 2016; Cumbaa et al., 2018) from the lower and middle Favel Formation (Keld Member) horizons analyzed in this study, a central subprovince distinct from northern one is further supported for late Cenomanian to early Turonian time (Kilmury & Brink, 2022).

### Preservational potential

The low apparent diversity of the Laurier Limestone bed assemblage (12 apparent species; Table 2) relative to that of the Marco Calcarenite assemblage (25 apparent species; Table 2) appears to represent an ecologically stressed community and supports the stratigraphic position of the Cenomanian-Turonian OAE2 event near the base of the Laurier Limestone beds of the Keld Member (Kyser et al., 1993). Since diversity sampling coverage tends to be highest in faunal assemblages associated with more calcareous lithologies (Kilmury & Brink, 2022), the excellent fossil preservation conditions of the Laurier Limestone beds should in theory have more complete coverage relative to the poorer preservational conditions of the ‘Near Keld base, Vermilion R.’ horizon associated with calcareous mudstone, though its apparent diversity is nearly identical with only nine apparent species observed from ‘Near Keld base, Vermilion R.’ and significantly lower than that of the Marco Calcarenite assemblage (Table 2). Together the higher preservation potential and low apparent diversity of the Laurier Limestone bed assemblage relative to the ‘Near Keld base, Vermilion R.’ and Marco Calcarenite microvertebrate assemblages provides further support for the stratigraphic position of the OAE2 event near the base of the Laurier Limestone beds in addition to the organic δ^13^C excursion observed by Kyser et al. (1993). The OAE2 stratigraphic position within the upper Keld Member may also explain why there appears to be significantly more macrovertebrate occurrences and higher apparent diversity in the Keld Member compared to the Assiniboine Member (Table 1).

## Supplemental Information

10.7717/peerj.15493/supp-1 Supplemental Information 1 Specimen of Vertebrate A under fluorescent light.

10.7717/peerj.15493/supp-2 Supplemental Information 2 *Apsopelix anglicus* (MM V-157).

10.7717/peerj.15493/supp-3 Supplemental Information 3 *Heterohelix globulosa*.

10.7717/peerj.15493/supp-4 Supplemental Information 4 *Cimolichthys* sp. (MGS 106-15-15-1).

10.7717/peerj.15493/supp-5 Supplemental Information 5 *Pachyrhizodus minimus* (MM V-3212).

10.7717/peerj.15493/supp-6 Supplemental Information 6 Recently donated (A and B) and previously collected (C) macrofossils from the Favel Formation of MB.

10.7717/peerj.15493/supp-7 Supplemental Information 7 Presence-absence analysis.

10.7717/peerj.15493/supp-8 Supplemental Information 8 Data for presence-absence analysis.

10.7717/peerj.15493/supp-9 Supplemental Information 9 Favel microvertebrate catalogue.

10.7717/peerj.15493/supp-10 Supplemental Information 10 Vertebrae measurements.

## Abbreviations

**Institutional abbreviations**

CMN: Canadian Museum of Nature, Ottawa, Ontario
FDM: Fort Dauphin Museum, Dauphin, MB
MGS: Manitoba Geological Survey, Winnipeg, MB
MM: Manitoba Museum, Winnipeg, MB (previously Manitoba Museum of Man and Nature)
ROM: Royal Ontario Museum, Toronto, Ontario
RSM: Royal Saskatchewan Museum, Regina, Saskatchewan
UM: University of Manitoba, Department of Earth Sciences, Winnipeg, MB

**Anatomical abbreviations**

ang: angular
b: basioccipital
cl: cleithrum
co: coracoid
de: dentary
f: frontal
la: lacrimal
mc: mesocoracoid
met: metapterygoid
ms: mesethmoid
mx: maxilla
n: nasal
op: opercle
pa: parietal
pf: prefrontal
pfin: pectoral fin
pmx: premaxilla
po: preopercle
psph: parasphenoid
pt: posttemporal
q: quadrate
sc: scapula
smx: supramaxilla
so: subopercle
v: vertebra
vo: vomer

## Additional Information and Declarations

### Field Study Permissions

The following information was supplied relating to field study approvals (*i.e*., approving body and any reference numbers): Field work was done under MB Heritage Permits A41-20 and A36-21 issued by the Government of Manitoba Historic Resources Branch.

### Data Availability

The following information was supplied regarding data availability: The raw data (list of specimens examined and vertebral measurements) are available in the Supplemental Files.
