# Supplementary figures and images for: Microvertebrate faunal assemblages of the Favel Formation (late Cenomanian-middle Turonian) of Manitoba, Canada
Source: PeerJ. 2023 Aug 3;11:e15493. doi: 10.7717/peerj.15493 (PMC10404398; doi:10.7717/peerj.15493)

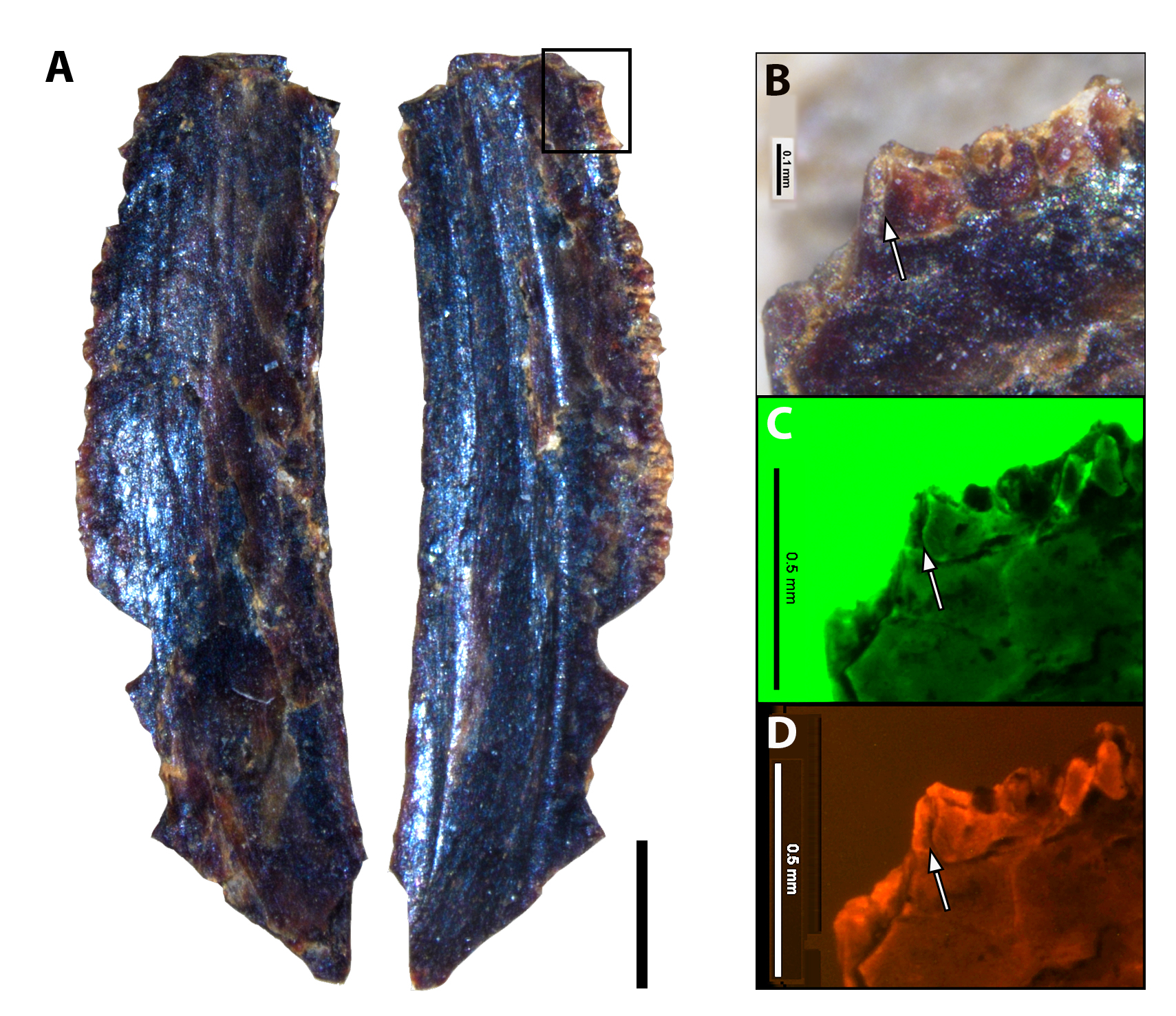

Supplement: Supplemental Information 1 [file peerj-11-15493-s001.jpg]

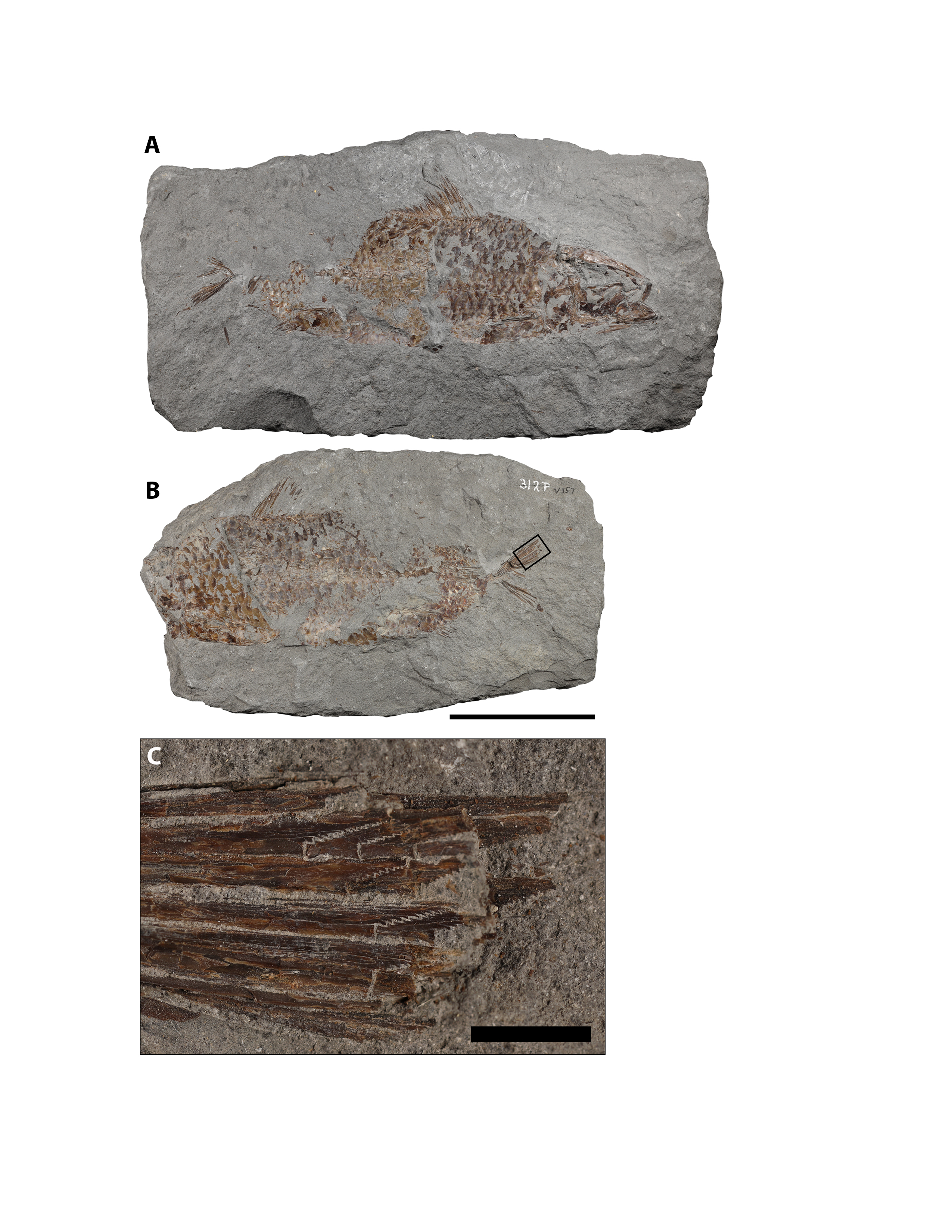

Supplement: Supplemental Information 2 [file peerj-11-15493-s002.jpg]

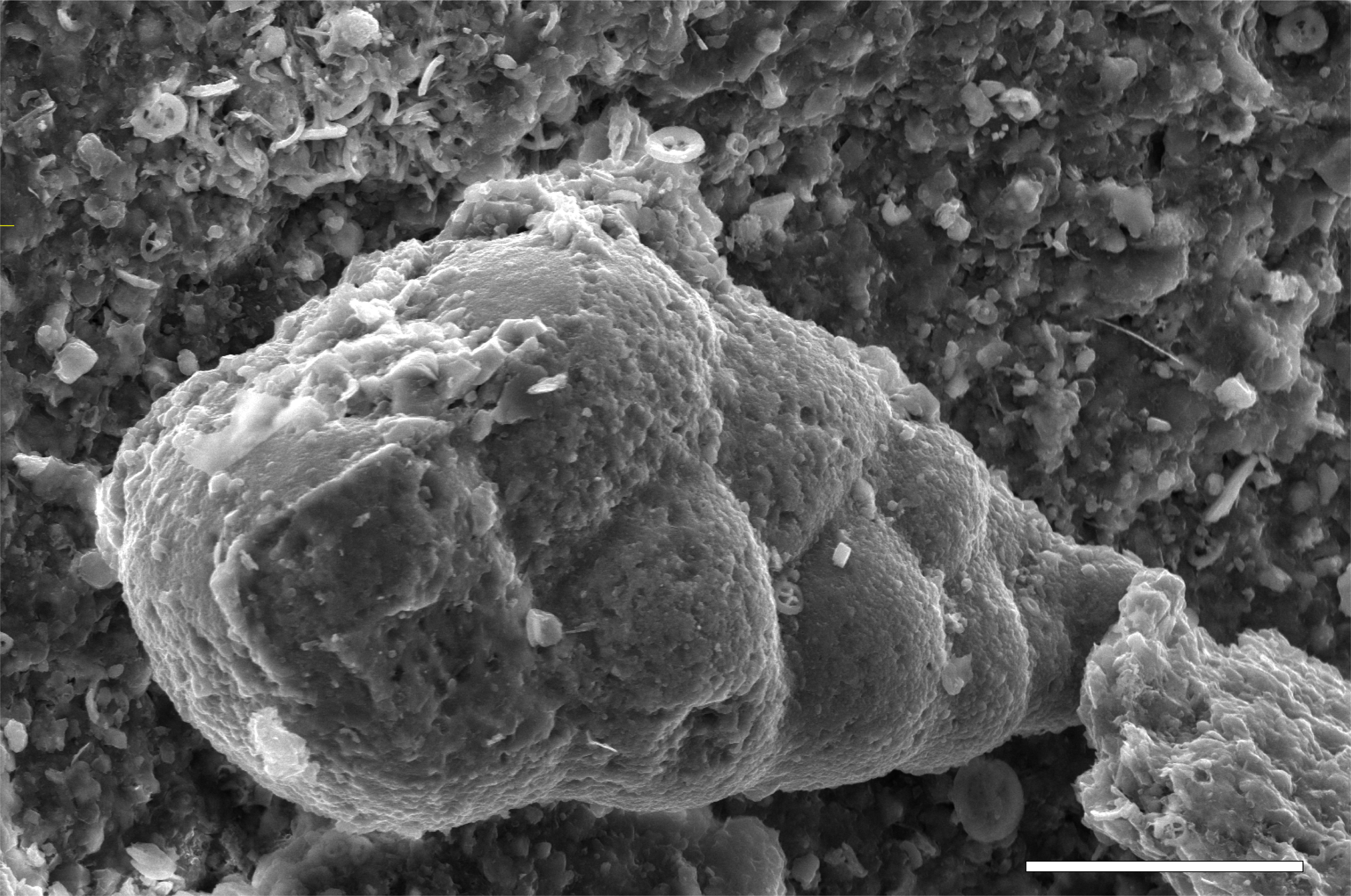

Supplement: Supplemental Information 3 [file peerj-11-15493-s003.jpg]

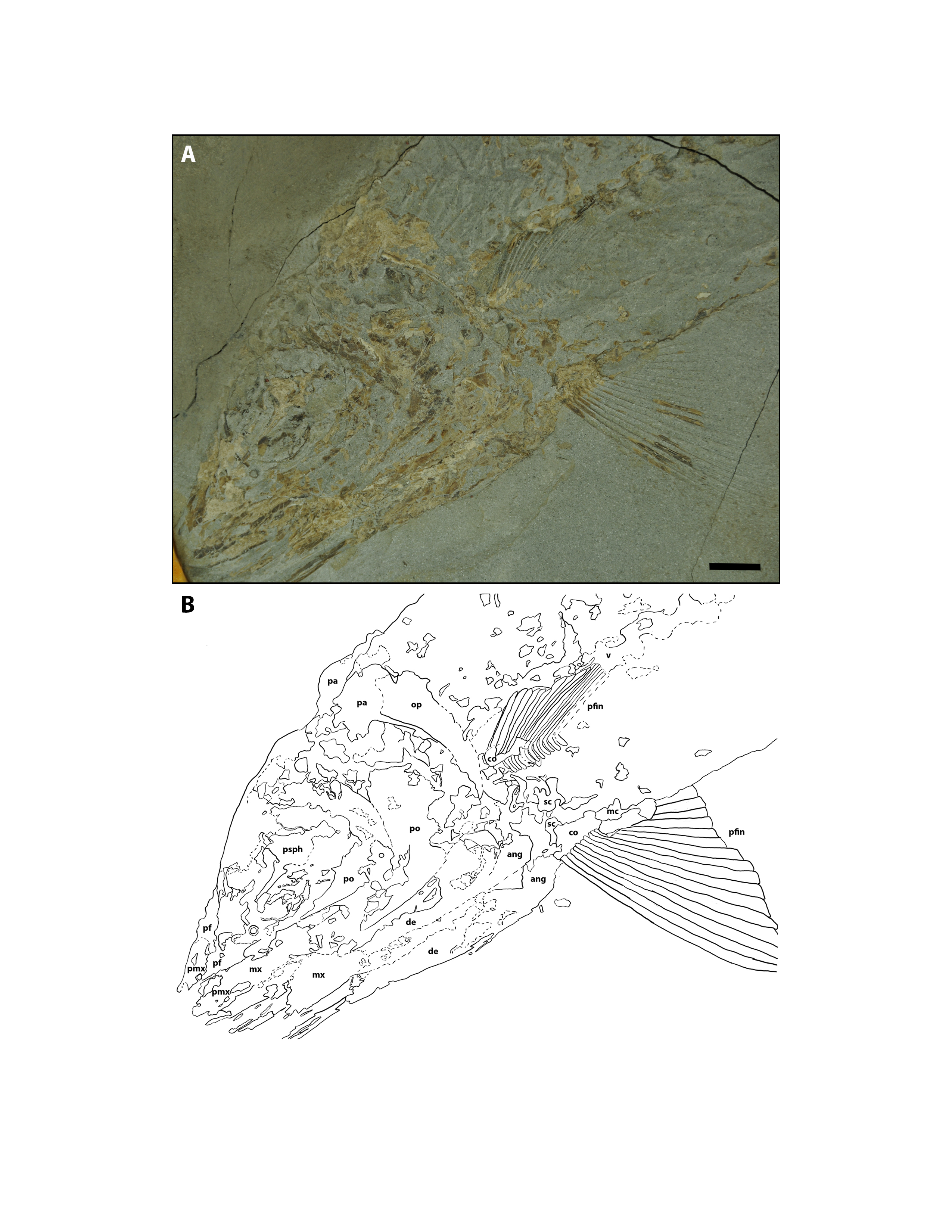

Supplement: Supplemental Information 4 [file peerj-11-15493-s004.jpg]

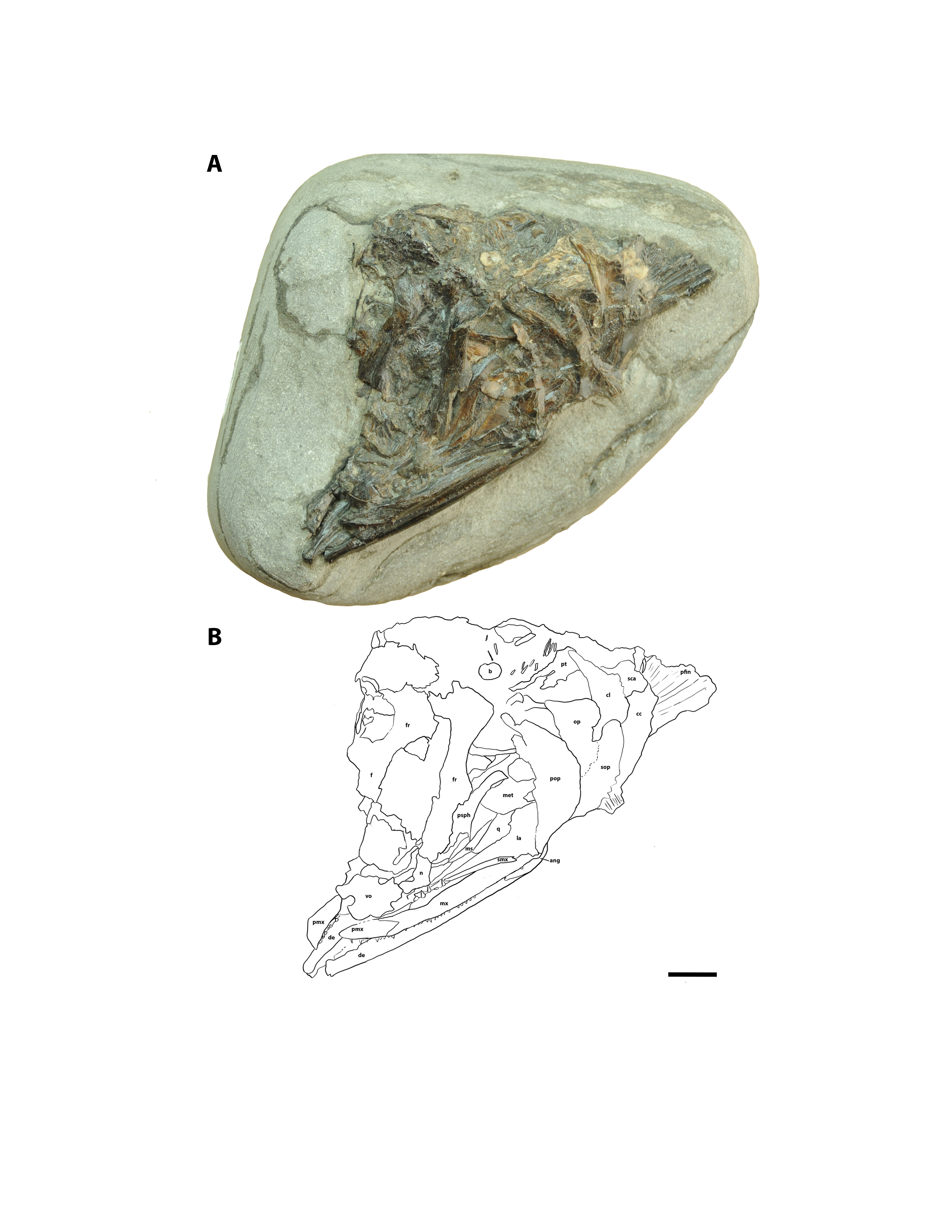

Supplement: Supplemental Information 5 [file peerj-11-15493-s005.jpg]

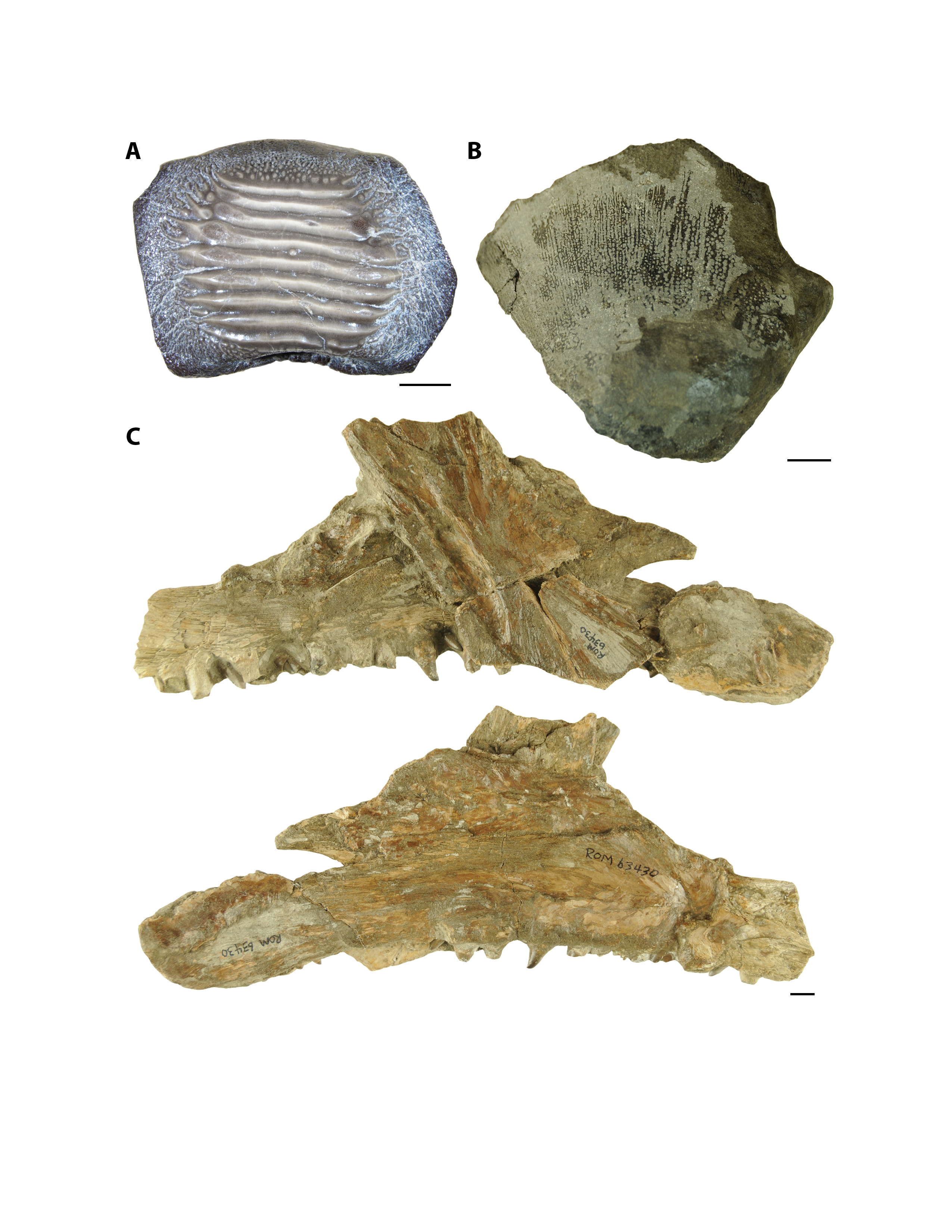

Supplement: Supplemental Information 6 [file peerj-11-15493-s006.jpg]
