# Supplementary material for: Microvertebrate faunal assemblages of the Favel Formation (late Cenomanian-middle Turonian) of Manitoba, Canada
Source: PeerJ. 2023 Aug 3;11:e15493. doi: 10.7717/peerj.15493 (PMC10404398; doi:10.7717/peerj.15493)
Supplement: Supplemental Information 7 [file peerj-11-15493-s007.docx]

**Table S1. Presence-Absence analysis of the late Cenomanian to early Turonian Western Interior Seaway relative to the MB escarpment.** Faunal occurrences were obtained from the following sources: MB escarpment from Table 3 and Kilmury (2022); Watino, Alberta (AB) from Fox, (1984), Wilson & Chalifa (1989), and Cook et al. (2013); Lac de Bois, Northwest Territories (NWT) from Cumbaa et al. (2018); South Dakota (SD) from Kilmury& Brink (2022); Kansas (KS) from Bice & Shimada (2016), McIntosh, Shimada, & Everhart (2016), and Kilmury & Brink (2022); and Texas (TX) from Kilmury & Brink (2022).

| **Taxon** | **MB Escarpment** | **Watino, AB** | **Lac de Bois, NWT** | **SD** | **KS** | **TX** |
| --- | --- | --- | --- | --- | --- | --- |
| **Actinopterygii** |  |  |  |  |  |  |
| *Albula* sp. undet. |  |  |  |  |  | **x** |
| *Ananogmius* sp. |  |  |  |  | **x** |  |
| *Anomoeodus* sp. |  |  |  |  | **x** | **x** |
| *Apateodus* sp. | **x** | **x** |  |  | **x** |  |
| *Apsopelix anglicus* | **x** |  |  |  | **x** | **x** |
| *Aquilopiscis wilsoni* |  |  | **x** |  |  |  |
| *Avitosmerus canadensis* |  |  | **x** |  |  |  |
| *Bananogmius aratus* |  |  |  |  | **x** |  |
| *Bananogmius ellisensis* |  |  |  |  | **x** |  |
| *Bananogmius* sp. undet. |  |  |  | **x** | **x** | **x** |
| *Bardackichthys carteri* |  |  |  |  |  | **x** |
| *Belonostomus* cf. *B. longirostris* |  | **x** |  |  | **x** | **x** |
| *Boreiohydrias dayi* |  |  | **x** |  |  |  |
| *Cimolichthys* cf. *C. levesiensis* | **x** | **x** | **x** |  |  |  |
| *Coelodus streckeri* |  |  |  |  | **x** |  |
| *Coelorhynchus* sp. undet. |  |  |  |  |  | **x** |
| *Cumbaaichthys oxyrhynchus* |  |  | **x** |  |  |  |
| *Cylindracanthus* sp. undet. |  |  |  |  | **x** |  |
| *Dercetoides* sp. |  | **x** |  |  |  |  |
| *Elopopsis* sp. | **x** |  |  |  |  |  |
| *Enchodus gladiolus* |  |  |  |  | **x** |  |
| *Enchodus petrosus* | **x** |  |  |  | **x** |  |
| *Enchodus shumardi* | **x** | **x** |  |  | **x** | **x** |
| *Enchodus* sp. undet. | **x** |  | **x** | **x** | **x** | **x** |
| *Gillicus arcuatus* | **x** | **x** |  |  | **x** |  |
| *Grypodon* sp. undet. |  |  |  |  |  | **x** |
| *Gyrodus* sp. |  |  |  |  | **x** |  |
| *Hadrodus* sp. |  |  |  |  | **x** | **x** |
| *Holocentroides* sp. |  |  |  |  | **x** |  |
| *Ichthyodectes ctenodon* | **x** | **x** | **x** |  | **x** | **x** |
| *Laminospondylus transversus* |  |  |  |  |  | **x** |
| *Lepidotes mantelli* |  |  |  |  |  | **x** |
| *Leucichthyops* sp. |  | **x** |  |  |  |  |
| *Macropoma* sp. |  |  |  |  | **x** |  |
| *Micropycnodon kansasensis* | **x** |  |  |  | **x** |  |
| *Ornatipholis sahtu* |  |  | **x** |  |  |  |
| ?*Osmeroides* cf. *O. delicatus* |  | **x** |  |  |  |  |
| ?*Osmeroides* cf. *O. transversus* |  | **x** |  |  |  |  |
| *Osmeroides* sp. |  | **x** | **x** |  |  |  |
| *Pachyrhizodus caninus* |  |  |  |  | **x** |  |
| *Pachyrhizodus minimus* | **x** |  |  |  | **x** | **x** |
| *Pachyrhizodus* sp. undet. |  |  |  | **x** | **x** | **x** |
| *Paramicrodon estesi* |  |  |  |  | **x** |  |
| *Pentanogmius* sp. undet. |  |  |  |  | **x** |  |
| *Plethodus* sp. |  |  |  |  |  | **x** |
| *Protosphyraena* sp. | **x** |  |  | **x** | **x** | **x** |
| *Pycnodus* sp. undet. |  |  |  |  |  | **x** |
| *Saurodon* sp. undet. |  |  |  |  |  | **x** |
| *Syllaemus* sp. undet. |  |  |  | **x** |  |  |
| *Thrissopater intestinalis* |  |  |  |  | **x** |  |
| *Thryptodus loomisi* | **x** |  |  |  |  | **x** |
| *Thryptodus zitteli* |  |  |  |  | **x** |  |
| *Xiphactinus lowii* |  |  |  |  | **x** |  |
| *Xiphactinus* sp. | **x** | **x** |  | **x** | **x** | **x** |
| **Chondrichthyes** |  |  |  |  |  |  |
| *Anomotodon sp.* |  |  |  |  | **x** |  |
| *Archaeolamna* ex. gr. *kopingensis* | **x** | **x** |  |  | **x** |  |
| *Cantioscyllium decipiens* |  |  |  |  | **x** |  |
| *Carcharias amonensis* |  |  |  | **x** | **x** |  |
| *Carcharias sasktatchewanensis* |  |  |  | **x** | **x** |  |
| *Carcharias* aff. *C. striatula* |  | **x** |  |  |  |  |
| *Carcharias tenuiplicatus* |  |  |  | **x** | **x** |  |
| *Carcharias woodbinensis* |  |  |  |  |  | **x** |
| *Carcharias* sp. undet. |  |  |  | **x** |  | **x** |
| *Cardabiodon* cf. *ricki* | **x** | **x** |  |  | **x** |  |
| *Chiloscyllium greeni* |  |  |  |  | **x** |  |
| *Corax* sp. undet. |  |  |  |  |  | **x** |
| *Cretalamna* ex. gr. *appendiculata* | **x** | **x** |  | **x** | **x** | **x** |
| *Cretalamna serrata* |  |  |  |  |  | **x** |
| *Cretalamna* sp. undet. | **x** | **x** |  |  | **x** | **x** |
| *Cretodus crassidens* |  |  |  |  | **x** | **x** |
| *Cretodus semiplicatus* |  | **x** |  |  | **x** | **x** |
| *Cretodus* sp. undet. | **x** |  |  | **x** | **x** | **x** |
| *Cretomanta canadensis* | **x** |  |  | **x** | **x** |  |
| *Cretoxyrhina denticulata* | **x** | **x** |  | **x** | **x** | **x** |
| *Cretoxyrhina oxyrhinoides* |  |  |  |  |  | **x** |
| *Cretoxyrhina* sp. undet. |  |  |  |  |  | **x** |
| *Dallasiella willistoni* |  | **x** |  |  | **x** |  |
| *Dasyatis* sp. undet. |  |  |  |  |  | **x** |
| *Galeocerdo* sp. undet. |  |  |  |  |  | **x** |
| *Galeorhinus* sp. undet. |  |  |  |  |  | **x** |
| *Hybodus* sp. |  | **x** |  |  |  |  |
| *Ischyrhiza* cf. *I. mira* | **x** |  |  |  | **x** |  |
| *Ischyrhiza texana* | **x** |  |  |  | **x** |  |
| *Ischyrhiza* sp. undet. |  |  |  |  |  | **x** |
| *Isurus* sp. undet. |  |  |  |  |  | **x** |
| *Johnlongia parvidens* |  | **x** |  |  | **x** |  |
| *Lamna bicuspidatus* |  |  |  |  |  | **x** |
| *Lamna crassidens* |  |  |  |  |  | **x** |
| *Lamna falcatus* |  |  |  |  |  | **x** |
| *Lamna semiplicatus* |  |  |  |  |  | **x** |
| *Lamna* sp. undet. |  |  |  |  |  | **x** |
| *Leptostyrax crassidens* |  |  |  |  | **x** |  |
| *Leptostyrax macrorhiza* |  |  |  |  |  | **x** |
| *Leptostyrax* sp. undet. |  |  |  |  |  | **x** |
| *Meristodonoides rajkovichi* |  | **x** |  |  |  |  |
| *Meristodonoides* sp. |  |  |  |  | **x** |  |
| *Microscyliorhinus* sp. | **x** |  |  |  |  |  |
| *Odontaspis amonensis* |  |  |  |  | **x** |  |
| *Odontaspis parvidens* |  |  |  | **x** | **x** |  |
| *Odontaspis saskatchewanensis* | **x** |  |  |  | **x** |  |
| *Odontaspis subulata* |  |  |  |  | **x** |  |
| *Odontaspis watinensis* |  | **x** |  |  | **x** |  |
| *Odontaspis* sp. |  |  |  |  | **x** | **x** |
| *Onchopristis dunklei* |  |  |  |  | **x** |  |
| *Palaeoanacorax pawpawensis* | **x** | **x** |  | **x** | **x** | **x** |
| *Paranomotodon* sp. |  |  |  |  | **x** |  |
| *Polyacrodus illingsworthii* |  |  |  |  |  | **x** |
| *Polyacrodus* sp. |  | **x** |  |  |  |  |
| *Pseudocorax grantii* |  |  |  |  |  | **x** |
| *Pseudocorax laevis* |  |  |  |  | **x** |  |
| *Pseudocorax* sp. |  |  |  |  | **x** |  |
| *Pseudohypolophus mcnultyi* |  |  |  |  |  | **x** |
| *Ptychodus anonymus* |  | **x** |  | **x** | **x** | **x** |
| *Ptychodus decurrens* |  |  |  |  | **x** | **x** |
| *Ptychodus janevaii* |  |  |  |  | **x** |  |
| *Ptychodus latissimus* |  |  |  |  |  | **x** |
| *Ptychodus mammillaris* |  |  |  |  | **x** | **x** |
| *Ptychodus marginalis* | **x** |  |  |  | **x** |  |
| *Ptychodus mortoni* |  |  |  |  | **x** | **x** |
| *Ptychodus occidentalis* | **x** |  |  | **x** | **x** | **x** |
| *Ptychodus rhombodus* | **x** |  |  |  | **x** |  |
| *Ptychodus rugosus* | **x** |  |  |  |  | **x** |
| *Ptychodus whipplei* |  |  |  | **x** | **x** | **x** |
| *Ptychodus* sp. | **x** | **x** |  | **x** | **x** | **x** |
| *Ptychotrygon hooveri* |  |  |  |  |  | **x** |
| *Ptychotrygon* sp. |  |  |  |  | **x** |  |
| *Rhinobatos incertus* | **x** | **x** |  | **x** | **x** |  |
| *Rhinoptera* sp. undet. |  |  |  |  |  | **x** |
| *Roulletia canadensis* | **x** |  |  |  |  |  |
| *Scapanorhynchus* aff. *S. raphiodon* | **x** | **x** |  | **x** | **x** |  |
| *Scapanorhynchus texanus* |  |  |  |  |  | **x** |
| *Scapanorhynchus* sp. undet. |  |  |  |  |  | **x** |
| *Scyliorhinus arlingtonensis* |  |  |  |  | **x** |  |
| *Squalicorax curvatus* | **x** |  |  | **x** | **x** | **x** |
| *Squalicorax deckeri* |  |  |  |  | **x** |  |
| *Squalicorax falcatus* | **x** | **x** |  | **x** | **x** | **x** |
| *Squalicorax obliquus* |  |  |  |  |  | **x** |
| *Squalicorax parvidens* |  |  |  |  | **x** |  |
| *Synodontaspis liliae* | **x** |  |  |  |  |  |
| *Telodontaspis agassizensis* | **x** |  |  |  | **x** |  |
| **Reptilia** |  |  |  |  |  |  |
| *Adocus* sp. undet. |  |  |  |  |  | **x** |
| *Brachauchenius lucasi* |  |  |  |  | **x** |  |
| *Coniasaurus crassidens* |  |  |  |  | **x** | **x** |
| *Coniasaurus* sp. undet. |  |  |  |  |  | **x** |
| *Dallasaurus turneri* |  |  |  |  |  | **x** |
| *Deltasuchus motherali* |  |  |  |  |  | **x** |
| *Desmatochelys lowii* |  |  |  |  | **x** |  |
| *Elasmosaurus* sp. undet. |  |  |  |  |  | **x** |
| ?*Libonectes morgani* | **x** |  |  |  |  |  |
| *Megacephalosaurus eulerti* |  |  |  |  | **x** |  |
| *Mosasaurus* sp. |  |  |  |  | **x** |  |
| *Plesiosaurus* sp. |  |  |  | **x** | **x** | **x** |
| *Polycotylus latipinnis* | **x** |  |  |  |  |  |
| *Polyptychodon hudsoni* |  |  |  |  |  | **x** |
| *Polyptychodon interruptus* |  |  |  |  |  | **x** |
| *Polyptychodon* sp. undet. |  |  |  |  |  | **x** |
| *Protostega* sp. undet. |  |  |  |  |  | **x** |
| *Pteranodon* sp. |  |  |  | **x** |  | **x** |
| *Terminonaris robusta* | **x** |  |  |  |  |  |
| *Thalassomedon dentonensis* |  |  |  |  |  | **x** |
| *Toxochelys* sp. |  |  |  |  | **x** | **x** |
| *Trinacromerum bentonianum*? | **x** |  |  |  | **x** |  |
| Crocodilia indet. |  |  |  |  |  | **x** |
| Mosasauridae indet. | **x** |  |  | **x** | **x** | **x** |
| Plesiosauria indet. | **x** |  |  | **x** | **x** | **x** |
| Pliosauridae gen. et sp. nov. | **x** |  |  |  |  |  |
| Pterosauria indet. |  |  |  |  | **x** | **x** |
| Testudines indet. | **x** |  |  | **x** | **x** | **x** |
| **Aves** |  |  |  |  |  |  |
| cf. *Ichthyornis* sp. | **x** | **x** |  |  | **x** |  |
| Aves undet. |  |  |  |  |  | **x** |
| **Vertebrata undet.** |  |  |  |  |  |  |
| Vertebrate A | **x** |  |  |  |  |  |
| **Total No. Genera** | 38 | 28 | 9 | 21 | 61 | 53 |
| **Total No. Species** | 49 | 32 | 9 | 29 | 95 | 88 |
| **Total No. of Shared Genera** | - | 16 | 3 | 14 | 27 | 15 |
| **Total No. of Shared Species** | - | 17 | 2 | 14 | 32 | 17 |
| **Coefficient of Community, Genera** | - | 0.484848485 | 0.127659574 | 0.47 | 0.5 | 0.33 |
| **Coefficient of Community, Species** | - | 0.419753086 | 0.068965517 | 0.36 | 0.4 | 0.25 |
